# Canady Helios Cold Plasma Induces Breast Cancer Cell Death by Oxidation of Histone mRNA

**DOI:** 10.3390/ijms22179578

**Published:** 2021-09-03

**Authors:** Xiaoqian Cheng, Saravana R. K. Murthy, Taisen Zhuang, Lawan Ly, Olivia Jones, Giacomo Basadonna, Michael Keidar, Yasmine Kanaan, Jerome Canady

**Affiliations:** 1 Jerome Canady Research Institute for Advanced Biological and Technological Sciences, Takoma Park, MD 20912, USA; drxcheng@jcri-abts.com (X.C.); drsmurthy@jcri-abts.com (S.R.K.M.); drtzhuang@jcri-abts.com (T.Z.); llawan@jcri-abts.com (L.L.); ozjones@jcri-abts.com (O.J.); 2 School of Medicine, University of Massachusetts, Worcester, MA 01605, USA; giacomo.basadonna@umassmed.edu; 3 Department of Mechanical and Aerospace Engineering, The George Washington University, Washington, DC 20052, USA; keidar@gwu.edu; 4 Microbiology Department, Howard University, Washington, DC 20060, USA; ymkanaan@howard.edu; 5 Howard University Cancer Center, Howard University, Washington, DC 20060, USA; 6 Department of Surgery, Holy Cross Hospital, Silver Spring, MD 20910, USA

**Keywords:** breast cancer, cold atmospheric plasma, cancer treatment, oxidation of RNA, histone gene

## Abstract

Breast cancer is the most common cancer among women worldwide. Its molecular receptor marker status and mutational subtypes complicate clinical therapies. Cold atmospheric plasma is a promising adjuvant therapy to selectively combat many cancers, including breast cancer, but not normal tissue; however, the underlying mechanisms remain unexplored. Here, four breast cancer cell lines with different marker status were treated with Canady Helios Cold Plasma™ (CHCP) at various dosages and their differential progress of apoptosis was monitored. Inhibition of cell proliferation, induction of apoptosis, and disruption of the cell cycle were observed. At least 16 histone mRNA types were oxidized and degraded immediately after CHCP treatment by 8-oxoguanine (8-oxoG) modification. The expression of DNA damage response genes was up-regulated 12 h post-treatment, indicating that 8-oxoG modification and degradation of histone mRNA during the early S phase of the cell cycle, rather than DNA damage, is the primary cause of cancer cell death induced by CHCP. Our report demonstrates for the first time that CHCP effectively induces cell death in breast cancer regardless of subtyping, through histone mRNA oxidation and degradation during the early S phase of the cell cycle.

## 1. Introduction

Breast carcinomas can be categorized into different entities based on clinical behavior, histologic features, and/or by biological properties, which is important for the discovery of novel treatments, the study of tumor evolution, and the identification of mechanisms of treatment resistance [1]. Tumors with expression of either the estrogen receptor (ER) or progesterone receptor (PR) in at least 1% of tumor cells are categorized as hormone receptor-positive (HR^+^) [2], and intrinsic subtypes of breast cancer are classified into luminal A (ER^+^/PR^+/−^/HER2^−^), luminal B (ER^+^/PR^+/−^/HER2^+/−^), basal-like (ER^−^/PR^−^/HER2^−^), and HER2-positive (ER^−^/PR^−^/HER2^+^) [3,4]. Systemic and local treatments are addressed by three major breast cancer subtypes: HR^+^HER2^−^, HER2^+^, and TNBC [5]. About 70% of breast cancer cases are HR^+^HER2^−^ [6] and endocrine agents are used to down-regulate ER signaling. HER2^+^ breast cancer constitutes 15–20% of cases, and HER2-targeted therapy includes anti-HER2 antibodies such as trastuzumab and pertuzumab, and small-molecule tyrosine kinase inhibitors such as lapatinib and neratinib. Basal-like (TNBC) makes up approximately 15% of breast cancer cases [6] and its molecular pathophysiology remains poorly understood.

Cold atmospheric plasma (CAP) as a novel anti-cancer therapy across various cancer types has been investigated for over a decade. A variety of different CAP devices have been developed, and their significant anti-cancer capabilities over 20 cancer types *in vitro*, [7] including breast cancer [8,9,10,11,12,13], have been tested. The efficacy of CAP treatment on breast cancer has been demonstrated both *in vitro* and *in vivo*; possible pathways include activation of MAPK/JNK and NF-kB pathways [9], and inhibition of STAT3 and IL-6R pathways [13] by reactive oxygen species. However, the differential effects of CAP on breast cancers of different molecular subtypes have yet to be reported, except in our previous study [12]. Canady Helios Cold Plasma (CHCP) was developed at JCRI-ABTS (U.S. Patent No. 9.999,462-19, June 2018 [14]) and has been demonstrated to effectively eliminate various types of solid tumor including breast carcinoma [12,15,16]. The first clinical trial using CAP for the treatment of cancer in the United States was approved for CHCP by the Food Drug Administration (FDA) and received by the Jerome Canady Research Institute for Advanced Biological and Technological Sciences (JCRI-ABTS) and US Medical Innovations, LLC (USMI). A phase I FDA investigational device exemption trial by CHCP is currently underway to study the safety of the system.

The mechanism for the selectivity of CAP-induced cell death in cancerous cells rather than normal cells, by generating reactive species in the effluent and medium [17], remains largely unexplored. Some non-compendious evidences such as induction of oxidative stress through the Srx-Nrf2 antioxidant system [18], sestrin2-mediated nitric oxide synthase signaling [19], or CAP-induced epigenetic alterations [20] have been discussed, but none have emphasized the role of RNA oxidation in CAP-induced cell death. RNA functions both as a structurally integral part of [21], and in the regulation of, chromatin [22]. The association of negatively charged DNA to positively charged histone proteins is collectively called chromatin. Histone proteins play crucial roles in all cellular processes that involve chromosomal DNA, such as DNA replication, transcription, repair and recombination, and chromosome segregation [23,24]. Their assembly into chromosomes and their biosynthesis are tightly regulated by DNA synthesis during the S phase of the cell cycle. Regulation occurs at both the transcriptional and post-transcriptional levels, leading to several-fold increases during the S phase [25,26,27,28,29] when regulatory proteins directly bind to subtype-specific regulatory elements (SSREs) in the promoter regions of histone genes [25,27,30,31,32]. Core histone overexpression outside of the S phase is toxic, and deregulation of histone genes leads to loss of chromosomes, resulting in DNA damage and cell death [33,34,35,36,37]. Accumulation of mutations in genes that control cell proliferation eventually leads to a cancerous state. The uncontrolled cell proliferation of the cancerous state requires an orchestrated regulation of histone gene expression (H1, H2A, H2B, H3, and H4), which is tightly regulated during the S phase [25,29] of the cell cycle [27,30,32].

The aim of this study is to prove that CHCP is a precise therapy for different subtypes of breast cancer and to reveal its anti-cancer mechanism. Four breast cancer cell lines including MCF-7 (luminal A, ER^+^/PR^+^/HER2^−^), BT-474 (luminal B, ER^+^/PR^+^/HER2^+^), MDA-MB-231 (basal-like, ER^−^/PR^−^/HER2^−^), and SK-BR-3 (ER^−^/PR^−^/HER2^+^) were tested with CHCP at various dosages. In this study we report that degradation of histone RNA in breast cancer cells by CHCP treatment leads to cell death, which is the key differentiating factor for the selective induction of cell death in breast cancer cells.

## 2. Results

The cold plasma device used in this study, CHCP, has been described in detail in our previous study [16]. Briefly, the device is a combined system of an electrosurgical generator and a conversion unit which up-converts voltage up to 4 kV, down-converts frequency to less than 300 kHz, and down-converts power to less than 40 W. The combined system is paired with a Canady Helios Cold Plasma Scalpel for the delivery of the plasma beam.

**Figure 1:**
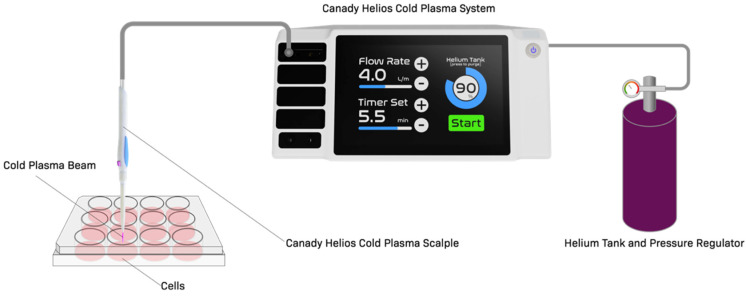
Schematic image of Canady Helios Cold Plasma setup for the treatment of breast cancer cells.

A schematic image of the device for the treatment of breast cancer cells *in vitro* is shown in Figure 1.

### 2.1. Proliferation Reduction after CHCP Treatment

Breast cancer cell lines with different receptor status showed power- and time-dependent reduction in cell viability (reported in previous study [12]) and proliferation rate (Supplementary Figure S1) after CHCP treatment. Increasing treatment time and power led to lower cell viability and slower growth rate across all 4 cell lines, among which SK-BR-3 was the most sensitive and BT-474 required the strongest dosage. This provides an overview of each breast cancer subtype’s reaction to CHCP treatment under different settings.

**Figure 2:**
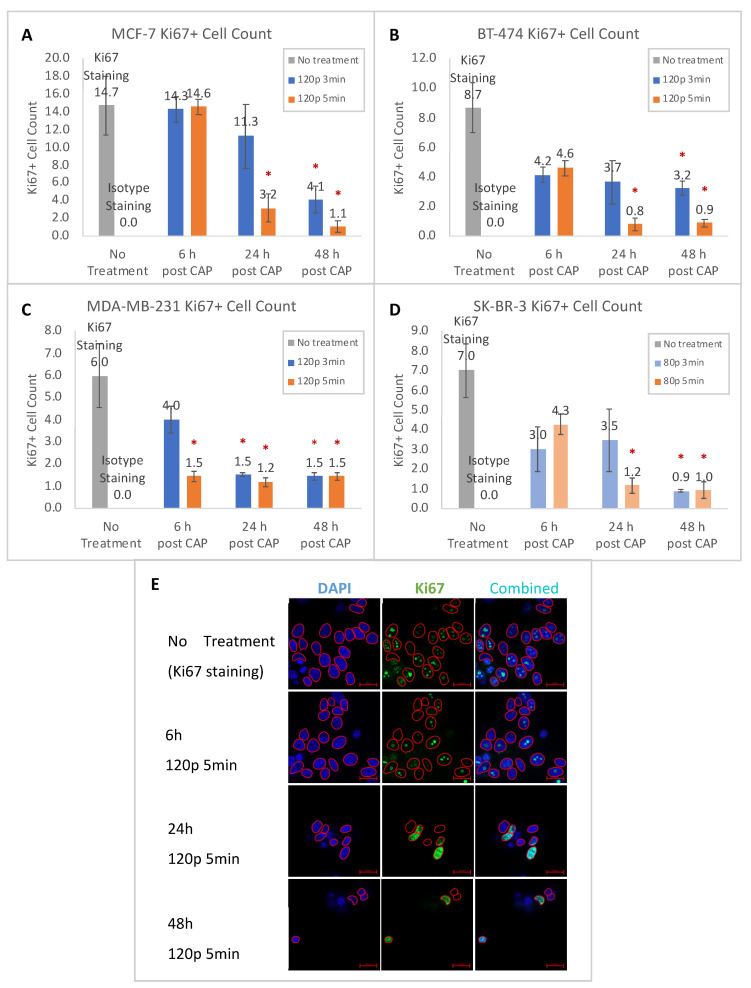
Averaged quantification plot of ‘Ki-67^+^’ cell counts of (**A**) MCF-7, (**B**) BT-474, (**C**) MDA-MB-231, and (**D**) SK-BR-3 cells 6/24/48 h post-CHCP treatment at 120 or 80 P for 3 or 5 min. (* above the bars denotes statistical significance of CAP-treated group compared to NT. * *p* < 0.05). NT = No Treatment. (**E**) Representative confocal microscopic images of MCF-7 cells. NT or 6/24/48 h post-CAP treatment at 120 P for 5 min (Alexa Fluor 488 conjugated Ki-67 Rabbit mAb probe was shown in green, DAPI for nucleus staining was shown in blue. Scale bar 20 μm).

Cytotoxicity and reduction in cell proliferation by CHCP in breast cancer cells were visualized by a Ki-67 marker. The number of Ki-67^+^ cells was reduced by CHCP treatment in all four breast cancer cell lines in a power- and time-dependent manner (Figure 2A–D). Over 6, 24, and 48 h incubations post-CHCP treatment, Ki-67^+^ cell number gradually decreased. In addition to the reduced Ki-67^+^ cell count, the shape and size of the nuclei changed after CHCP treatment. Shrinkage and fragmentation were seen in all cells treated with the highest dosage of CHCP (representative images for MCF-7 as an example are shown in Figure 2E).

Ki-67 staining was remarkably brighter in cells with NT or with lower dosages of CHCP than those treated with higher dosages (Supplementary Figures S2–S5). Taking MCF-7 as an example, Ki-67 was seen throughout the nucleoplasm, and in most cases in co-localization with nucleoli in NT samples. At low-dose or short incubation time (6 h of 120 P for 3 min and 5 min, 24 h of 120 P for 3 min, and 48 h of 120 P for 3 min), nucleoli were intact and Ki-67 expression was still observed but in fewer cells; at high dose (24 h and 48 h of 120 P for 5 min), Ki-67 staining was either diminished or presented throughout the nuclei with disrupted nucleoli. The same pattern was seen in all four breast cancer cell lines with slightly different treatment conditions. For MCF-7, BT-474, and MDA-MB-231, CHCP treatment at 120 P for 5 min caused visual damage to the nuclei after 24 h incubation. For SK-BR-3, visual nuclear damage was observed as early as 6 h post-CHCP treatment at a lower dose of 80 P for 3 min.

### 2.2. Apoptosis Induced by CHCP Treatment

**Figure 3:**
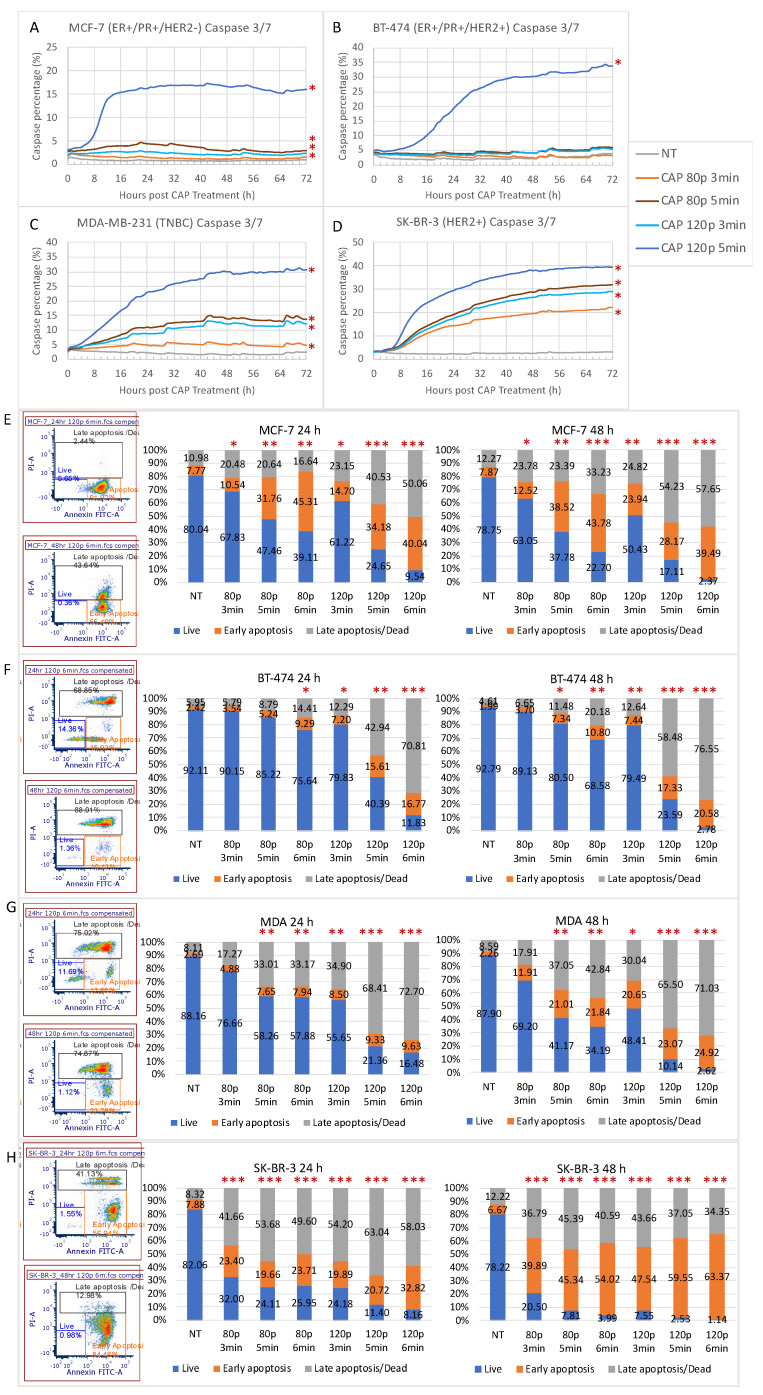
Caspase 3/7 activity of breast cancer cell lines at 0–72 h post-CAP treatment (**A**–**D**) Quantification of caspase 3/7 activity of MCF-7, BT-474, MDA-MB-231, and SK-BR-3 over 72 h post-CHCP treatment by IncuCyte Live-Cell Imaging. (**E**–**H**) Representative images and averaged quantification plots of ‘live’, ‘early apoptosis’, and ‘late apoptosis/dead’ populations of MCF-7, BT-474, MDA-MB-231, and SK-BR-3 at 24 and 48 h post-CHCP treatment by flow cytometry. (* above the bars denotes statistical significance of CAP-treated group compared to corresponding No Treatment group. * *p* < 0.05, ** *p* < 0.01, and *** *p* < 0.001).

Caspase 3/7 activity was measured to determine whether CHCP merely reduced cell proliferation or induced cell death, as well as to visualize the timeline of apoptotic activities for each cell line. Representative phase-contrast images and quantification of caspase activity for select time points after CHCP treatment (Supplementary Figures S6–S9 and Figure 3A–D respectively) were in accordance with the viability data shown above. Compared to NT, significant apoptosis activity was observed in MCF-7, MDA-MB-231, and SK-BR-3 cells by all CHCP treatment doses, with SK-BR-3 being the most susceptible and MCF-7 the most resistant. BT-474 cells were the most robust among all the cell lines, but the highest dosage (120 P for 5 min) was able to result in statistically significant apoptosis. For all four breast cancer cell lines, apoptosis (if induced by CHCP) initiated within 4 h post-treatment and plateaued after 48 h post-treatment. The slope of the caspase 3/7 curve representing the kinetics of apoptotic activity is cell line- and dose-dependent, suggesting that the higher the CHCP dose, the earlier and faster apoptosis occurred.

Cell apoptosis at 24 and 48 h post-CHCP treatment was confirmed by flow cytometry. Representative scatter and quantification plots of ‘live’, ‘early apoptosis’, and ‘late apoptosis/dead’ cells for four breast cancer cell lines were shown in Supplementary Figures S10–S13 and Figure 3E–H. For all cell lines, in the NT samples, ~80–90% of cells were viable (‘Live’, Annexin V^−^/PI^−^). In contrast, exposure to CHCP treatment for 3, 5 or 6 min at 80 P or 120 P induced apoptosis to various extents over 24 and 48 h incubation times. The significant decrease in the population of ‘live’ and increase in ‘early apoptosis’ cells with increasing treatment power and time compared to NT were seen in all cell lines (*p* value ranges from < 0.05 to < 0.001, Figure 3E–H). SK-BR-3 was the most susceptible to CHCP treatment, and BT-474 was the most resistant among the four cell lines. Strongly statistically significant different (*** *p* < 0.001) ‘live’ populations were observed 24 h post-CHCP treatment in SK-BR-3 with the lowest treatment dosage (80 P for 3 min). BT-474 did not show significant decreases until 24 h with CHCP treatment at 80 P for 6 min (* *p* < 0.05) or 48 h with CHCP treatment at 80 P for 5 min (* *p* < 0.05).

Comparing the quantification of 48 to 24 h post-CHCP treatment, more cells entered apoptosis between 24 and 48 h (observed in all cell lines for all treatment conditions), indicating that CHCP-induced apoptosis could take up to 48 h.

### 2.3. Temporal Progress of the Cell Cycle

**Figure 4:**
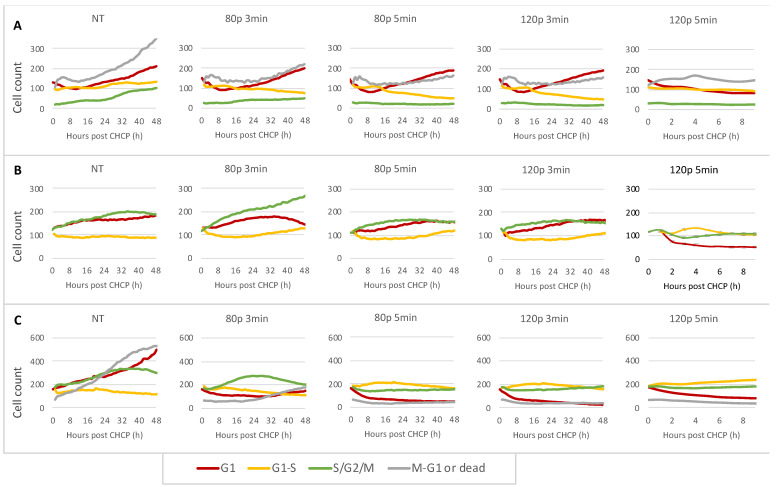
Quantification of (**A**) MCF-7, (**B**) BT-474, and (**C**) MDA-MB-231 cells in G1, G1-S, S/G2/M, and M-G1 phases over 72 h after CHCP treatment at various dosages.

Quantification data for cells in the G1 (Red), S/G2/M (Green), or S to G1 transition (S-G1, Yellow) phases are shown in Figure 4. Confluence of stable cell lines after CHCP treatment and phase-contrast images (Supplementary Figures S14–S16) consistent with the confluence of non-transfected cell lines (Supplementary Figure S1) confirmed that cell function was not altered by the lentivirus.

For MCF-7 cells treated with low doses of CHCP (80 P for 3 min and 5 min, and 120 P for 3 min, Figure 4A), during the first 8 hours post-treatment the number of cells in the G1 phase decreased drastically while the number of cells in the G1-S transition phase and S/G2/M phase did not have significant changes. The cell population in all phases decreased when treated with the highest dose of CHCP (120 P for 5 min), where most cells died within 9 h (Supplementary Figure S14); thus, quantification was only plotted for 9 h. 

Low doses of CHCP (80 P for 3 min and 5 min, and 120 P for 3 min) did not result in significant changes to the cell cycle of BT-474 (Figure 4B), which corresponds with caspase 3/7 activity. With the highest CHCP dose of 120 P for 5 min, where apoptosis was observed, the number of cells in G1 and S/G2/M drastically decreased whereas the G1-S transition phase slightly increased, suggesting that the cell cycle did not progress from the G1 to the S/G2/M phase completely.

For MDA-MB-231 (Figure 4C), low doses of CHCP (80 P for 3 min and 5 min, 120 P for 3 min) quickly decreased the number of cells at the G1 phase within 8 h, but a limited number of cells progressed towards the S/G2/M phases (yellow and green). High doses of CHCP (120 P for 5 min) induced apoptosis immediately; therefore, the number of cells in all phases decreased.

### 2.4. Whole Transcriptome Analysis

**Figure 5:**
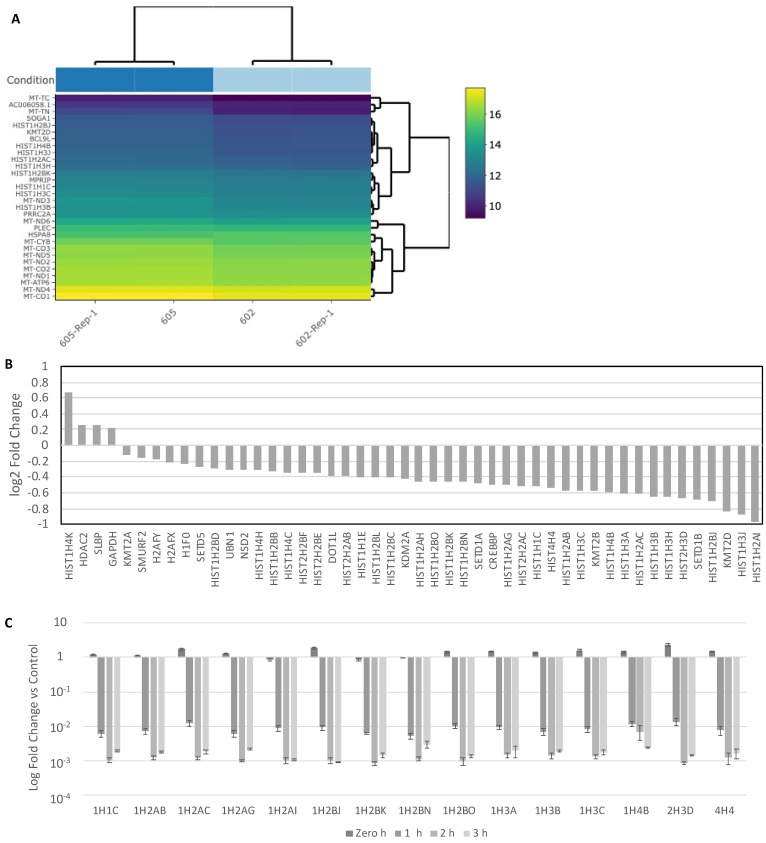
(**A**) Heatmap of the top 30 differentially expressed genes between CHCP-treated MDA-MB-231 cells (602) and mocks (605). Gene expression levels are displayed on a log (absolute values) scale. (**B**) Next-generation sequencing (NGS) whole-transcriptome analysis in fold-changes (log2 scale) of histone and histone-related transcripts after 6 h CHCP treatment in MDA-MB-231 cells. (**C**) Histone mRNA degradation after CHCP treatment. Fold-change of histone mRNA after 0, 1, 2 and 3 h post-treatment compared to mock control samples of MDA-MB-231 cells.

RNA sequencing technology has gained prominence for high-throughput transcriptome analysis in recent years [38]; many breast cancer RNA transcriptomics studies have prognostic markers as well as novel therapeutic targets [39,40,41]. However, breast cancer RNA transcriptomics after CAP treatment has not been investigated. Therefore, to reveal the potential genes and mechanisms involved in CHCP-induced cell death, we used an NGS approach with Illumina HiSeq as a means of examining and comparing the transcriptomes of CHCP-treated breast cancer cell lines. A total of 45,037,559 and 37,683,510 reads, for mock (control) and CHCP-treated TNBC cell line MDA-MB-231 samples respectively, were sequenced. This cell line represents the four cell lines because it responded with a moderate amount of resistance to CHCP as shown above. Gene expression of mock MDA-MB-231 cells was used as a baseline for CHCP-treated cells. From a total of 25,671 mRNA genes, 75 were differentially expressed (44 up-regulated and 35 down-regulated) between the mock control and CHCP-treated samples. The GO analysis for the significantly differentially expressed genes were clustered by their gene ontology, and the enrichment of gene ontology terms was tested using the Fisher exact test (GeneSCF v1.1-p2). Supplementary Table S1 shows the gene ontology terms, if any, that are significantly enriched with an adjusted *p*-value of less than 0.05 in the differentially expressed gene sets. With manual methods of categorization of differentially expressed genes, histone transcripts were very prominent among different classes of gene transcripts such as zinc finger proteins, long non-coding RNAs, and kinases (Figure 5A). The 24 histones and histone-related gene transcripts were differentially expressed in the CHCP-treated samples compared to mocks (Figure 5B).

To further analyze the differential expression of histone genes in CHCP-treated MDA-MB-231 cells, the top 15 highly down-regulated histone gene transcripts were chosen, and their expression levels were compared among 0, 1, 2 and 3 h post-CHCP treatment by qRT-PCR (Figure 5C). These four time points were arbitrarily chosen to assess the initial expression of histone genes after CAP treatment. Down-regulation of histone mRNA initiated as soon as the first hour of incubation following CHCP treatment. The downregulation of all 15 histone mRNAs was several-fold lower than the fold-changes observed by NGS. Statistical analyses for the down-regulated genes *HIST1H1C, HIST1H2AB, HIST1H2AC, HIST1H2AG, HIST1H2AI, HIST1H2BJ, HIST1H2BK, HIST1H2BN, HIST1H2BO, HIST1H3A, HIST1H3B, HIST1H3C, HIST1H3H, HIST1H4B, HIST2H3D, HIST4H4* are shown in Supplementary Figure S17. The only histone mRNA which did not show statistical significance compared to controls was *HIST1H3C*; however, the general trend was similar to other histone mRNAs. None of these mRNAs showed statistical significances between 1 h to 3 h groups, unlike the zero-hour group (*p* < 0.01). Except for mRNAs *HIST1H2BN, HIST1H2BK, HIST1H2AI*, all other histone mRNAs were generally up-regulated at the zero-hour incubation time point compared to controls; *HIST4H4* (*p* < 0.01) and *HIST1H2BJ* (*p* < 0.01) showed significance. It was interesting that all histone mRNAs are highly downregulated in the CHCP-treated groups after one hour incubation, suggesting a distinct mechanism for the emergence of histone-related cell death induced by CHCP. Thus, qPCR validation of histone mRNA down-regulation prompted us to further explore the mechanism of CHCP-induced cell death.

### 2.5. Oxidation of RNA after CHCP Treatment 

**Figure 6:**
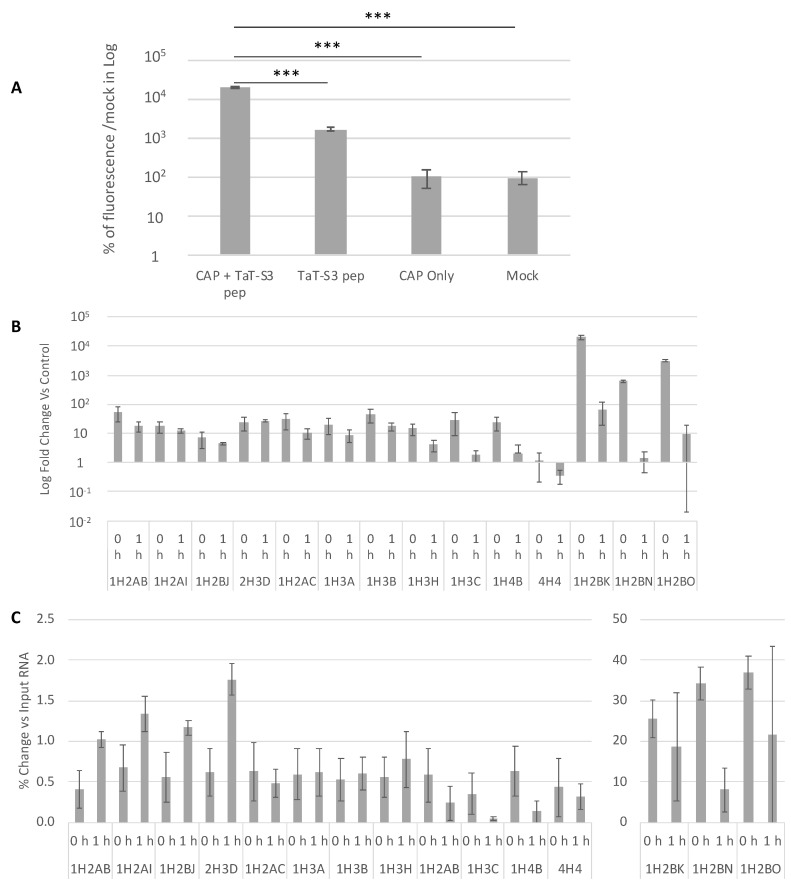
(**A**) The fluorescence intensity of RNA oxidation probed in situ with fluorescence-tagged 8-oxoG binding peptide probe on CHCP-treated or untreated live MDA-MB-231 cells. The average SEM of the ratios is plotted. ANOVAs were used followed by post-hoc comparisons using Student’s *t-*test with Bonferroni corrections as appropriate. *** *p* < 0.001. (**B**) Pull-down of 8-oxoG histone RNA; the fold-change of immunoprecipitated 8-oxoG histone RNA after 0 and 1 h post-CAP treatment compared to immunoprecipitated 8-oxoG mock control samples of MDA-MB-231 cells. (**C**) The percentage change of 8-oxoG modification in histone genes at zero-hour and one-hour incubation after CAP treatment in MDA-MB-231 cells between the CAP-8-oxoG immunoprecipitated group and input group.

Recent studies have shown that CAP-generated reactive oxygen and nitrogen species (RONS) could induce 8-oxoguanine (8-oxoG) formation in nucleic acids [42]. To investigate the in situ 8-oxoG modification of RNA after CHCP treatment, we modified and generated a previously designed [43] human ribosomal protein S3 (hRpS3) peptide probe (TaT-S3-peptide) that binds tightly to 8-oxoG sites on RNA. Fluorescence signals were compared between CHCP-treated and untreated cells (Figure 6A), probed or not probed with TaT-S3-peptide (ANOVA for CAP-TaT-S3, mock-TaT-S3, CAP, and mock groups: F[3,44] = 587.78, *p* < 4.3 × 10^−33^). The average fluorescence signal in the CAP-TaT-S3 group and mock-TaT-S3 group were 20410% and 1738% respectively compared to unprobed CAP or mock groups (*t*-test *p* < 1.96 × 10^−11^). The CAP-TaT-S3 group was 1174% higher than mock-TaT-S3 group (*t*-test *p* < 1.36 × 10^−11^), even though there were only ~50% of cells compared to the mock-TaT-S3 control group because of cell death induced by CHCP. The results demonstrate that there were high incidences of 8-oxoG formation in the nuclei acids of CHCP-treated TNBC cells and that the down-regulation observed in RNASeq data and qRT-PCR analyses would be due to 8-oxoG modification of histone mRNAs.

To further investigate 8-oxoG formation after CHCP induction on histone mRNA specifically, one portion of total RNA from mock zero- (MZ) and one-hour (M1), and CHCP zero- (CP0) and one-hour (CP1) groups were used as inputs (MZ_IN_, M1_IN_, CP0_IN_, and CP1_IN_ respectively) and the other was used to isolate 8-oxoG-containing transcripts via immunoprecipitation with an anti-8-oxoG antibody (MZ_IP_, M1_IP_, CP0_IP_, and CP1_IP_ respectively), followed by qRT-PCR analysis of 16 histone gene transcripts (Figure 6B). CP0_IP_ and CP1_IP_ group samples had significantly higher quantities of all 16 histone genes compared to the MZ_IP_ and M1_IP_ groups (Supplementary Figure S18). There was significant (*p* < 0.01) mRNA amplification difference between groups CP0_IP_ vs. MZ_IP_, CP1_IP_ vs. M1_IP_, CP0_IP_ vs. MZ_IN_, CP1_IP_ vs. M1_IN_, and CP0_IP_ vs. CP1_IP_ among all the histone genes analyzed. *HIST1H3C* between CP1_IP_ vs. M1_IP_ (*p* < 0.014), *HIST4H4* between CP0_IP_ vs. MZ_IN_ (*p* < 0.012), and *HIST1H2BK, HIST1H2BN* and *HIST1H2BO* between CP1_IP_ vs. M1_IN_ (*p* < 0.065; 0.08; 0.09 respectively) were not significant but were significant between CP0_IP_ vs. CP1_IP_ (*p* < 0.0009; 0.003; and 0.005 respectively); however, the trends for these genes were similar. We quantified the percentage of 8-oxoG modification on histone RNA in CP0_IP_ and CP1_IP_ compared to the CP0_IN_ and CP1_IN_ groups (Figure 6C). The 8-oxoG modification events were significantly higher in CP0_IP_ and CP1_IP_ compared to CP0_IN_ and CP1_IN_ groups (*p* < 0.001) for all the histone genes analyzed. Taken together, our data provides evidence of 8-oxoG oxidative modification in these 16 histone mRNAs in CHCP-treated TNBC cells. Importantly, the negative fold-change in histone mRNA shown in Figure 5C is due to 8-oxoG modification and degradation of histone mRNA rather than regulation at the transcription level.

### 2.6. DNA Damage Induced by CHCP Comes after RNA Oxidation

**Figure 7:**
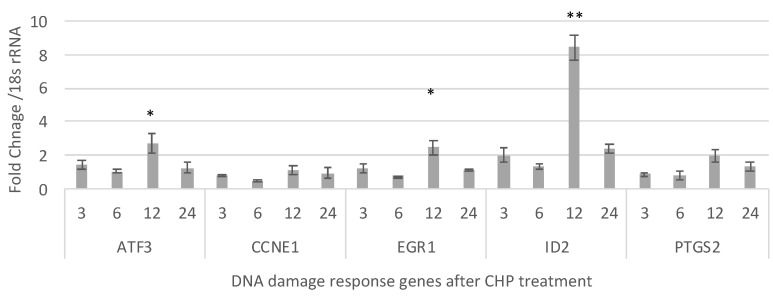
Fold-changes in DNA damage response gene transcripts *ATF3, CCNE1, EGR1, ID2* and *PTGS2* genes compared to mock controls in MDA-MB-231 cells post-CHCP treatment at different incubation time points (3, 6, 12, & 24 h). Statistical analysis was performed using Student’s *t*-test. * *p* < 0.005; ** *p* < 0.001.

The expression of the DNA damage response genes activating transcription factor 3 (*ATF3*) [45], cyclin E1 (*CCNE1*) [46], early growth response 1 (*EGR1*) [47], inhibitor of DNA binding 2 (*ID2*) and prostaglandin–endoperoxide synthase 2 (*PTGS2*) compared to mock groups were quantified to understand the mechanism of the primary contributor to cell death induced by CHCP treatment (Figure 7). These are early responders of DNA damage and we found significant up-regulation of *ATF3, EGR1*, and *ID2* genes transcripts (*p* < 0.05) by more than two-fold increases only after 12 h of incubation. *CCNE1* and *PTGS2* showed no significant up-regulation at any of the time points compared to mock controls. These results show that DNA damage is not the primary cause of CHCP-induced cell death in TNBC cells.

## 3. Discussion

In recent decades, studies on a variety of tumor cell types have been published using different CAP devices. CHCP has been previously demonstrated to be a promising anti-cancer treatment for several cancers including TNBC [15,16]. Molecular features and corresponding tumor subtypes of each breast tumor cell line are feasible models for tumors of the same subtype [4]. We are the first to analyze dose-dependent effects of CAP on breast cancer cell lines based on molecular subtypes using CHCP via monitoring of Ki-67 expression, apoptosis progression, and the cell cycle. Ki-67 expression is an independent prognostic parameter according to breast cancer molecular subtypes in breast cancer patients [48]. At the molecular level, expression, localization and post-translational modification of Ki-67 are regulated by the cell cycle [49]; during the early G1 phase, the Ki-67 antigen is located in the nucleoplasm, unlike its nucleolus location in the S and G2 phases [50], and is regulated differently in non-cancerous and cancerous cells [51], providing an insight into the mechanism of CAP selectivity towards cancerous cells. Our data on Ki-67 expression in four breast cancer subtypes indicate down-regulation of Ki-67 by CAP with slightly different dosages.

Apoptosis induction by CHCP treatment was dose-dependent, which was quantitatively and visually assayed by flow cytometry and by IncuCyte for each molecular subtype of breast cancer cell line. Apoptosis was seen earlier in cells treated with higher CHCP power and time. Previously, Park et al. reported that MDA-MB-231 (TNBC) underwent a higher rate of apoptosis and a decreased proliferation rate upon CAP treatment than MCF-7 (ER^+^/PR^+^/HER2^−^) cells [20], which is in agreement with our findings. In addition, HER2^+^ cell lines showed a more sensitive response while BT-474 (ER^+^/PR^+^/HER2^+^) were the most resistant to CHCP treatment. Temporal progression of cell cycle data demonstrates that the number of cells in G1 phases decreased quickly within the first 8 h after CAP treatment, but the increase in the G1-S transition phase or S/G2/M phase during this period was much smaller, if present, suggesting that the transition from the G1 to the S/G2/M phase was disrupted by CAP.

Cold atmospheric plasma as an anti-cancer therapy has been reported, either applied on its own [52], or in combination with chemotherapy [8] or radiation therapy [53]. The mechanism of CAP-induced cell death in malignant cells has not yet been established; however, some pointers have been discussed [54]. Several studies reporting phosphorylation of H2AX with CAP treatment in different cancer types have thus concluded that plasma-generated ROS induce DNA damage [55,56,57,58]. Recently, Bekeschus et al. [59] argued that DNA damage was a consequence rather than a cause for CAP-induced cell death. Our data is in agreement with their findings, demonstrating oxidation-enrichment of histone RNA transcripts in CAP-treated cells compared to control groups. Although RNA oxidation altered expression levels of many transcripts, this was particularly pronounced on transcripts involved in chromatin modification and the DNA-damage response. Our data showed that transcriptional activation of DNA damage early response genes *ATF3* [60], *EGR1* [61], and *ID2* [62] occurred only at 12 h after CAP treatment—a late response in DNA damage response genes. These observed alterations were considered as an indication of cellular responses rather than consequences of apoptosis and necrosis.

Moreover, cell cycle analysis after CHCP treatment revealed a significant decrease in the number of cells at G1 with a higher number of cells in S/G2/M phases with strong dosages when apoptosis activity was induced. This connotatively demonstrates that the machinery and components required for the S phase of the cell cycle to proceed were impaired or unavailable [63]. The ability of CAP treatment to induce specific 8-oxoG modification in RNA molecules and to subsequently compromise the stability of these modified histone transcripts and their associated pathways represents a potentially novel mechanism for the detrimental and apoptotic effects of CAP treatment. Growing evidence suggests that RNA oxidation might be involved in chromatin destabilization and cell death [64,65]. Primarily, studies have focused on RNA oxidation, degradation, and oxidative stress in neurological disorders [66,67]. CAP-induced RNA differential expression, such as *Sp1* [68], *ZNRD1*, and *ZNRD1-AS1* [69] has also been reported recently. We are the first to report that RNA oxidation is the primary cause of CAP-induced cell death in cancer cells. Studies have shown that 8-oxoG could base-pair with adenosine, hence mRNA oxidation can introduce amino acid point mutations [70,71] which lead to errors in translation and destabilization of proteins. It has also been revealed that mRNA oxidation could compromise its translational activity and fidelity, and induce several signaling pathways to mediate inflammatory responses and induce apoptosis [72]. The literature has also argued about 8-oxoG RNA oxidation and RNA stability [73]. RNA Seq data showed that histone mRNAs are predominantly differentially expressed, and qRT-PCR quantification suggests that 8-oxoG RNA modification of histone mRNA also leads to its degradation. Histones H2A and H2B are the key proteins in packaging of DNA into nucleosomes [74] and are replication-dependent and cell cycle-regulated, increasing 15 to 35-fold in the S phase during DNA replication [75,76]. Given that histone gene transcription is tightly regulated during the S phase of the cell cycle, any alterations in histones by RNA degradation or unstable proteins would be detrimental to chromatin stability and cell survival. This phenomenon plays an important role in the selectivity of CAP treatment on rapidly proliferating cancer cells rather than normal cells. CAP-induced histone RNA oxidation, degradation, and cell death would be the characteristic differentiating factor for the more frequently occurring S phase events in tumors than in normal cells. 

Furthermore, because of this aspect, breast cancer cell line subtypes required slightly different power or time settings to achieve 100% elimination. For example, rapidly proliferating TNBC MDA-MB-231 cells needed shorter CAP treatment time compared to relatively slow-paced BT-474 cells. Moreover, the expression levels of histone H2A and H2B variants have been shown to be varied among breast cancer cell line subtypes and were mediators of drug resistance [77]. This variation in histone mRNA would also lead to different CAP power and treatment time for the setup. Since there was a magnitude increase in 8-oxoG-binding peptide probe (TaT-S3-peptide) binding to total RNA in the CHCP-treated samples compared to controls, there is reason to believe CAP-induced 8-oxoG RNA modification is not selective to any transcript. However, the tight cell cycle-dependent transcriptional regulation of histone RNA makes CAP treatment particularly lethal to fast-proliferating cancer cells rather than normal cells, whereas other RNA transcripts could survive the CAP insults as they are not cell cycle-dependent and could be replenished without delay. Furthermore, in dividing cells, the half-life of histone mRNAs and their degradation are tightly regulated at the end of the S phase or when DNA replication is inhibited [78]. Given that CAP treatment induces 8-oxoG RNA modification and strand breaks in genomic DNA [42,59], ceasing of DNA replication could occur, making histone mRNA degradation even more pronounced. Even though CAP-induced 8-oxoG RNA modification is not selective to any transcript, 18s rRNA, used as normalization RNA (housekeeping gene) during qPCR experiments, did not show any down-regulation as the amplification cycles (CT values) remained highly constant at all the sampled time points with and without CHCP treatment. This could be because the 18s rRNA assemble within the associated ribosomal protein into functional units; this structural characteristic could make ribosomal RNA more stable to CAP insults compared to other RNA (“naked”) molecules. This phenomenon could also be true for other RNA molecules that are constantly bound to RNA-binding proteins until they undergo the natural cycle of degradation in the cell.

The histone mRNA *HIST1H2AB*, which was significantly enriched within the chromatin structure [79], has been shown to be up-regulated in breast cancer tissue and in colorectal cancer [80]. Histone mRNAs *HIST1H2AC, HIST1H2BF*, and *HIST1H2BO* are shown to be overexpressed in breast cancer cells, contributing to the stability of the nucleosome structure for increased proliferation of cells [81] and mediating the up-regulation of BCL2 expression to stimulated cell proliferation [82]. Studies have also shown that chemoresistance in human TNBC MDA-MB-231 cells was due to histone regulation, specifically the up-regulation of *HIST1H2BK* [83], *HIST1H3C*, and *HIST1H2AB* [84]. All the histone RNAs that we analyzed in this study have been shown to have variants that contribute to cancer development and progression [85]. High proliferation rates of cancer cells require coherent histone regulation, and any flaws would lead to chromatin instability and cell death. Taken together, our data provides evidence of oxidative modification in histone mRNA as the primary cause of CAP-induced cell death.

## 4. Materials and Methods

Breast cancer cell lines of 4 different subtypes were treated with cold plasma, and apoptosis was observed and quantified in all 4 cell lines. One of the 4 subtypes, triple-negative breast cancer MDA-MB-231, was used as a representative cell line to further investigate the anti-cancer mechanisms of cold plasma because it responded with a moderate amount of resistance to CHCP. All experiments were repeated at least 3 times with 2 replicates each time. All experiments were performed at the Jerome Canady Research Institute for Advanced Biological and Technological Sciences in Takoma Park, MD, USA.

### 4.1. Cold Plasma Device

Canady Helios Cold Plasma™ (CHCP) was developed at JCRI-ABTS (U.S. Patent No. 9,999,462 B2 June 2018 [14]). CAP was generated by the CHCP, which was reported in our previous publication [16]. CHCP parameters were set as follows: helium flow rate at 3 L/min; and power settings at 80 P (15.7 W), 100 P (22.3 W), and 120 P (28.7 W).

### 4.2. Cell Culture

Human breast ductal carcinoma BT-474 and breast adenocarcinoma SK-BR-3 were purchased from ATCC and cultured according to the provided protocol. Human adenocarcinoma cell lines MCF-7 and MDA-MB-231 were generously donated by Professor Kanaan’s laboratory at Howard University. SK-BR-3 was cultured with McCoy’s 5A and BT-474, MCF-7 and MDA-MB-231 were cultured with RPMI 1640 medium, both supplemented with 10% fetal bovine serum (FBS) (Sigma-Aldrich, St. Louis, MO, USA) and 1% Pen Strep (Thermo Fisher Scientific, Waltham, MA, USA). All cell lines were cultured at 37 °C in a 5% CO_2_ humidified incubator (Thermo Fisher Scientific). When reaching approximately 80% confluence, cells were seeded at a concentration of 10^5^ cells/well into 12-well plates (USA Scientific, Ocala, FL, USA) with 1 mL culture media per well for all assays.

### 4.3. IncuCyte Live Cell Imaging for Confluence, Caspase Activity, and Cell Cycle

Confluence of cells: Cells were seeded in 12-well culture plates and treated with CHCP as described above, and imaged with an IncuCyte S3 Live-Cell Analysis System (Sartorius, Aubagne, France) for up to 72 h. Student’s *t-*test was performed on each treatment dosage, and every hour post-CHCP treatment compared to No Treatment (NT) controls or mocks; * denotes statistical significance if *p* < 0.05 at 48 to 72 h post-treatment.

Caspase 3/7 activity: IncuCyte^®^ Caspase-3/7 Dyes for Apoptosis (Sartorius) couple the activated caspase-3/7 recognition motif (DEVD) to a DNA intercalating dye and are ideally suited to the mix-and-read, real-time quantification of cells undergoing caspase-3/7 mediated apoptosis. Addition of IncuCyte^®^ Caspase-3/7 Dyes to normal healthy cells is non-perturbing to cell growth and morphology. When added to tissue culture medium, the inert, non-fluorescent substrate crosses the cell membrane where it is cleaved by activated Caspase-3/7, resulting in the release of the DNA dye and fluorescent staining of nuclear DNA.

Breast cancer cells were seeded in 12-well plates at a density of 10^5^ cells/well, treated or untreated with CAP, followed by staining with IncuCyte Caspase 3/7 dye. Then the cells were placed in an IncuCyte and scanned with phase-contrast and green channels at 10 × magnification at intervals of 1 h for 72 h. After scanning, fluorescent objects were quantified using the IncuCyte integrated analysis software with background subtraction. The percentage of caspase-3/7-active cells out of the total population per image from 0 to 72 h post-CHCP treatment was plotted.

Cell cycle tracking: The IncuCyte^®^ Cell Cycle Green/Red Lentivirus Reagent (Sartorius) is a fluorescent, single cassette indicator expressing both GFP (green fluorescent protein) and mKate2 (red fluorescent protein) to distinguish between cells in the G1 and S/G2/M cell cycle phases without altering cell function. Stable cell populations for MCF-7, BT-474, and MDA-MB-231 cell lines were generated with the IncuCyte^®^ Cell Cycle Green/Red Lentivirus Reagent using puromycin selection. There was a technical difficulty when generating a stable cell population for HER2^+^ subtype SK-BR-3; attempts were made with another HER2^+^ cell line AU-565, but they also failed to produce a stable cell line with the IncuCyte^®^ Cell Cycle Green/Red Lentivirus Reagent. Therefore, cell cycle analysis was not performed on the HER2^+^ subtype.

Stable cells were seeded in 12-well plates at a density of 10^5^ cells/well, treated or untreated with CAP and placed in the IncuCyte for 3-day scanning. Cells were scanned every hour with phase-contrast, green, and red channels at 10 × magnification with a Cell-By-Cell Module for MCF-7 and MDA-MBA-231 cells. For BT-474 cells, which tend to grow in clusters, the Cell-By-Cell Module could not properly detect the boundaries between each cell, thus only basic scanning and analysis were used.

### 4.4. Flow Cytometry

Apoptosis assays were carried out on all 4 breast cancer cell lines with FITC Annexin V (BD Biosciences, Franklin Lakes, NJ, USA) and PI (ThermoFisher) by flow cytometry. Cells were seeded in 12-well plates and treated with CAP for various dosages. After incubation for the desired periods of time, cell culture media was removed, and cells were washed twice with PBS and detached with 250 μl of trypsin-EDTA (Sigma-Aldrich). Please note that the culture media, PBS for washing, and trypsin–EDTA were all collected and spun down for apoptotic staining and analysis. Plotting of data and analysis of results were performed with FCS Express (De Novo Software Version 7).

### 4.5. Confocal Microscopy

Immunofluorescent staining and imaging: Round cover glass (12 mm diameter, Fisher Scientific) was placed in 12-well plates and coated with fibronectin (Sigma-Aldrich) and collagen I (ThermoFisher) for at least 1 h prior to seeding cells. Seeding cells on cover slides in 12-well plates instead of chamber slides can produce a consistent CAP treatment effect with various dosages since the well size and cell number remain the same, as shown by the MTT viability assay and apoptosis assay by flow cytometry. After the CAP treatment and desired periods of incubation time, cells were stained with Alexa Fluor 488 conjugated Ki-67 Rabbit mAb (Cell Signaling Technology, Danvers, MA, USA) according to the manufacturer’s protocol. Briefly, cells were washed with phosphate buffered saline (PBS, ThermoFisher) and fixed with cold anhydrous methanol (pre-cooled in a −80 °C freezer) for 10 min at room temperature. After the methanol was aspirated, cells were washed twice with PBS and blocked in blocking buffer for 60 min. Then, Ki-67 and Isotype Control (Cell Signaling Technology) antibodies were diluted 1:200 with antibody dilution buffer—400 μL of which was added to designated wells. Cells were incubated overnight at 4 °C in a refrigerator protected from light. Round cover slides with cells were washed twice before they were carefully moved onto 1″ × 3″ × 1 mm microscope slides. The cells were first covered with Antifade Mounting Reagent with DAPI (Vector Laboratories, Burlingame, CA, USA) drops and then a 24 × 50 mm cover glass (Cancer Diagnostics, Durham, NC, USA), and were allowed to cure for up to 2 nights in the 4 °C refrigerator.

Breast cancer cells were treated with the desired CHCP dosages and Ki-67/DAPI co-staining was performed 6, 24, or 48 h post-CHCP treatment. Cells were imaged with a 63 × lens on a Confocal LSM 510 (Carl Zeiss, Jena, Germany) with 405 and 488 nm laser bands.

Intensity quantification: The intensity of Ki-67 expression was quantified using Zen Lite 3.1 from Zeiss. Nuclei were outlined with a spline contour tool in each image (image size: 108.36 μm × 108.36 μm, scale bar 50 μm). The average intensity of Ki-67 staining of each nucleus within the outline was measured by Zen Lite 3.1 and exported to and plotted by Microsoft Excel. Five images were analyzed for each condition, and in cases where there were no cells remaining after CAP treatment, the cell count was recorded as 0. For quantification, Ki-67-positive (Ki-67^+^) cell count was used instead of Ki-67^+^ cell percentage because the late apoptotic or dead cells were washed off during the staining process, resulting in a false total cell count. Nuclei that were clearly in focus were outlined and their mean fluorescence intensity (MFI) in the Ki-67 channel was recorded. The mean of Ki-67 MFI was calculated for each treatment group (5 images), including No Treatment and isotype controls. A Ki-67^+^ cell was defined as having its Ki-67 MFI greater than the lowest mean of the MFI of all groups, other than isotype controls, for each cell line.

### 4.6. RNA-Seq Library Preparation 

rRNA depletion and HiSeq sequencing: MDA-MB-231 cells were CAP-treated at 120 P (28.7 W) for 5 min according to the method mentioned above and incubated for 6 h. Total RNA was isolated by a guanidinium–phenol (TRIzol)-based kit (Zymo Research, Irvine, CA, USA) with double DNase digestion following the manufacturer’s instructions. RNA samples were quantified using a Qubit 2.0 Fluorometer (Life Technologies, Carlsbad, CA, USA) and RNA integrity was checked with a 4200 TapeStation (Agilent Technologies, Palo Alto, CA, USA). A total of 97.8% and 98.2% of reads were mapped on the ENSEMBL Homo sapiens reference genome for mock and treated sample cells respectively, and only unique reads that fell within exon regions were counted.

rRNA depletion was performed using a Ribozero rRNA Removal Kit (Illumina, San Diego, CA, USA). RNA sequencing library preparation used an NEBNext Ultra RNA Library Prep Kit for Illumina by following the manufacturer’s recommendations (NEB, Ipswich, MA, USA). Briefly, enriched RNAs were fragmented for 15 min at 94 °C. First-strand and second-strand cDNA were subsequently synthesized. cDNA fragments were end-repaired and adenylated at their 3′ ends, and a universal adapter was ligated to the cDNA fragments, followed by index addition and library enrichment with limited cycle PCR. Sequencing libraries were validated using the Agilent Tapestation 4200 (Agilent Technologies, Palo Alto, CA, USA), and quantified by using a Qubit 2.0 Fluorometer (Invitrogen, Carlsbad, CA, USA) as well as by quantitative PCR (Applied Biosystems, Carlsbad, CA, USA).

The sequencing libraries were multiplexed and clustered on 1 lane of a flowcell and loaded on the Illumina HiSeq instrument according to the manufacturer’s instructions. The samples were sequenced using a 2 × 150 paired end (PE) configuration. Image analysis and base calling were conducted by HiSeq Control Software (HCS). Raw sequence data (.bcl files) generated from Illumina HiSeq was converted into FASTQ files and de-multiplexed using Illumina’s bcl2fastq 2.17 software. One mismatch was allowed for index sequence identification.

Data analysis: After investigating the quality of the raw data, sequence reads were trimmed to remove possible adapter sequences and nucleotides of poor quality using Trimmomatic v.0.36. The trimmed reads were mapped to the Homo sapiens reference genome available on ENSEMBL using the STAR aligner v.2.5.2b. The STAR aligner is a splice aligner that detects splice junctions and incorporates them to help align entire read sequences. BAM files were generated as a result of this step. Unique gene hit counts were calculated by using featureCounts from the Subread package v.1.5.2. Only unique reads that fell within exon regions were counted. After extraction of gene hit counts, the gene hit counts table was used for downstream differential expression analysis. Using DESeq2, a comparison of gene expression between groups of samples was performed. The Wald test was used to generate *p*-values and Log2 fold changes. Genes with adjusted *p*-values < 0.05 and absolute log2 fold changes > 1 were identified as differentially expressed genes for each comparison. A gene ontology analysis was performed on the statistically significant set of genes by implementing GeneSCF software. The goa_human GO list was used to cluster the set of genes based on their biological processes and determine their statistical significance. A PCA analysis was performed using the “plotPCA” function within the DESeq2 R package. The plot shows the samples in a 2D plane spanned by their first two principal components. The top 500 genes, selected by highest row variance, were used to generate the plot.

### 4.7. Real-Time qPCR Validation

Real-time qPCR (qRT-PCR) was performed to further validate selected, differentially expressed histone RNAs from the NGS results. First-strand synthesis was carried out with 1 ug total RNA from CAP-treated and mock control MDA-MB-231 cells using random hexamer primers (for mRNA and lncRNA) with a reverse transcription SUPERSCRIPT IV 1ST STRND SYSTM (Thermo, USA), followed by qRT-PCR amplification of cDNA (50 ng) in a BIO-RAD CFX96 RealTime PCR Detection System (BIO-RAD, USA), using PowerUp™ SYBR™ Green Master Mix (Thermo, USA). Each sample was analyzed in triplicate. Internal control housekeeping gene 18 s rRNA was selected as described previously [86] and was used for normalization of all samples; subsequent fold-changes were calculated using the 2^−ΔΔCT^ method [87]. To detect DNA contamination, diluted RNA samples without first-strand synthesis were directly used for qPCR described above. Statistical analysis was performed using repeated measure ANOVAs followed by post-hoc comparisons using Student’s *t-*test with Bonferroni corrections as appropriate. *p* < 0.05 was considered statistically significant.

### 4.8. Oxoguanine (8-oxoG) Modification of RNA

Peptide synthesis: The Alexa 488-TaT-S3 peptide was synthesized by solid-phase peptide synthesis at ThermoFisher Scientific USA with the following sequence: [NH2]C (Alexa 488)GVLRFIMESGAKGSEVVVSGGGYGRKKRRQRRR[COOH]. The Alexa 488 dye was added to a cysteine on the N-terminus and conjugated using maleimide chemistry. The peptide was purified by high-performance liquid chromatography (HPLC) and freeze-dried after cleavage. The Alexa 488-TaT-S3 peptide was designed for the detection of 8-oxoguanine using a fluorophore-labeled probe with cell-penetrating ability as described previously [44]. The amino acid sequence “GVLRFIMESGAKGCEVVVSG” in the peptide is obtained from human ribosomal protein S3 (hRpS3) that tightly binds 8-oxoG sites. The peptide sequence YGRKKRRQRRR is from the transactivator (TAT) domain of the human immunodeficiency virus-1 (HIV-1) TAT protein that can effectively deliver proteins into cells and appears to not be limited by the size of the fusion protein. “GG” amino acids are the GG linker to bind TAT peptide to S3 peptide.

In situ detection of 8-oxoG RNA: MDA-MB-231 cells were seeded in 12-well plates at 10^5^/well and cultured at standard conditions for 24 h as described before. Alexa 488-TaT-S3 peptide at 1.0 μM final concentration was added to the cells and incubated for 2 h. CAP treatment at 120 P (28.7 W) for 5 min was carried out and cells were incubated for 24 h followed by washing with PBS. Fluorescence signals were read with a BioTek Synergy HTX (Winooski, VT, USA) microplate reader at 485 nm.

### 4.9. Pull-Down

RNA preparation: MDA-MB-231 cells were CAP-treated at 120 P (28.7 W) for 5 min according to the method mentioned above. One set of cells were processed immediately (zero-hour) and the other set were incubated for 1 h. Total RNA was isolated by a guanidinium–phenol (TRIzol)-based kit (Zymo Research Direct-zol) with triple DNase I digestion according to the manufacturer’s instructions (Zymo Research). Ribosomal RNA (rRNA) was depleted using a Ribo-Zero Gold rRNA Removal Kit (Illumina, San Diego, CA, USA) following the manufacturer’s instructions. Immunoprecipitations of 8-oxoG-containing RNA transcripts were performed as described previously [43] in biological triplicates for CAP-treated 0 (CP0) and 1 h (CP1), mock zero- (MZ) and one-hour (M1) conditions. Briefly, each sample was divided into portions and a portion of the input RNA was incubated with 10 μg of 8-oxo-7,8-dihydroguanosine (8-oxoG) monoclonal antibody (Trevigen, Gaithersburg, MD, USA) in immunoprecipitation (IP) buffer (10 mM Tris pH 7.4, 150 mM NaCl, 0.1% IGEPAL, and 200 units/mL SUPERaseIn RNA inhibitor (ThermoFisher Scientific)) in a 1 mL reaction volume for 4 h at 4 °C with rotation. The 8-oxoG antibody binds specifically to 8-oxoG-containing transcripts directly without mediation through an RNA-binding protein. SureBeads Protein A magnetic beads (Biorad, Hercules, CA, USA) were washed according to the manufacturer’s protocol and blocked in IP buffer supplemented with 0.5 mg/mL bovine serum albumin (BSA) for 2 h at room temperature. After being washed twice in IP buffer, the beads were resuspended in IP buffer, mixed with the RNA-antibody reaction and incubated for 2 h at 4 °C with rotation. Next, the beads were washed three more times in IP buffer before two rounds of competitive elution were performed. Each elution consisted of incubation of the beads with 100 μg of 8-oxo-dG (Cayman Chemical, Ann Arbor, MI, USA) in IP buffer for 1 h at 4 °C with rotation. The elution volume was then cleaned up using the RNA Clean and Concentrator-5 kit (Zymo Research) to isolate mock zero- (MZ_IP_) and one-hour (M1_IP_), and CAP zero- (CP0_IP_) and one-hour (CP1_IP_) RNA. First-strand synthesis was performed for 8-oxoG-immunoprecipitated and input RNA, followed by real-time qPCR (qRT-PCR) for histone RNAs.

### 4.10. Statistics

All assays were repeated at least 3 times and data was plotted by Microsoft Excel 2016 as the mean ± standard error of the mean. Data were analyzed by ANOVA; post-hoc comparisons were carried out using Student’s *t-*tests with Bonferroni corrections as appropriate. A *p* value < 0.05 was considered statistically significant. Differences were considered statistically significant for * *p* < 0.05, ** *p* < 0.01, and *** *p* < 0.001.

## 5. Conclusions

In this study, we report that CHCP is an effective treatment for all breast cancer subtypes, and the required dosage can be optimized based on receptor status. We are the first to show that 8-oxoG RNA oxidation of histone RNA followed by degradation and chromatin destabilization at the early S phase of the cell cycle is one key mechanism among others discussed in the literature for CAP-induced cell death. This mechanism is the key differentiating factor in Canady Helios Cold Plasma therapy-induced cancer cell death amongst four breast cancer cell lines, and sheds light on the mechanism for the selectivity of CAP treatment towards cancer cells without damaging normal tissue, as has been demonstrated by our ongoing research and by others. Based on our study, conditions and standards could be improved by precisely targeting this mechanism for advancing therapeutic inventions for breast and other cancers. Future *in vivo* studies on the effects of CHCP on cancer and normal human tissues are warranted for validation of the mechanisms and clinical applications of CHCP.

## Data Availability

All data and materials used in analysis are available upon reasonable request.

## Supplementary Materials

The following are available online at https://www.mdpi.com/article/10.3390/ijms22179578/s1, Figure S1: Confluence of breast cancer cells with or without CHCP treatment over 72 h; Figure S2: Ki-67 staining of MCF-7 (ER^+^/PR^+^/HER2−) cell line; Figure S3: Ki-67 staining of BT-474 (ER^+^/PR^+^/HER2^+^) cell line; Figure S4: Ki-67 staining of MDA-MB-231 (TNBC) cell line; Figure S5: Ki-67 staining of SK-BR-3 (HER2^+^) cell line; Figure S6: Phase-contrast images of MCF-7 (ER^+^/PR^+^/HER2−) cell line stained with Incucyte^®^ Caspase-3/7 Dyes for Apoptosis; Figure S7: Phase-contrast images of BT-474 (ER^+^/PR^+^/HER2^+^) cell line stained with Incucyte^®^ Caspase-3/7 Dyes for Apoptosis; Figure S8: Phase-contrast images of MDA-MB-231 (TNBC) cell line stained with Incucyte^®^ Caspase-3/7 Dyes for Apoptosis; Figure S9: Phase-contrast images of SK-BR-3 (HER2^+^) cell line stained with Incucyte^®^ Caspase-3/7 Dyes for Apoptosis; Figure S10: Apoptosis analysis of MCF-7 (ER^+^/PR^+^/HER2−) cell line; Figure S11: Apoptosis analysis of BT-474 (ER^+^/PR^+^/HER2^+^) cell line; Figure S12: Apoptosis analysis of MDA-MB-231 (TNBC) cell line; Figure S13: Apoptosis analysis of SK-BR-3 (HER2^+^) cell line; Figure S14: Cell cycle of MCF-7 (ER^+^/PR^+^/HER2−) stable cell line; Figure S15: Cell cycle of BT-474 (ER^+^/PR^+^/HER2^+^) stable cell line; Figure S16: Cell cycle of MDA-MB-231 (TNBC) stable cell line; Figure S17: Histone RNA degradation after CAP treatment statistical analysis; Figure S18: Pull-down of 8-oxoG Histone RNA statistical analysis; Table S1: The GO analysis for the significantly differentially expressed genes.
