# Supplementary material for: Canady Helios Cold Plasma Induces Breast Cancer Cell Death by Oxidation of Histone mRNA
Source: Int J Mol Sci. 2021 Sep 3;22(17):9578. doi: 10.3390/ijms22179578 (PMC8430908; doi:10.3390/ijms22179578)
Supplement: Supplementary file 1 [file ijms-22-09578-s001.zip › Sub_Supplementary Figures.pdf]

Supplementary Data

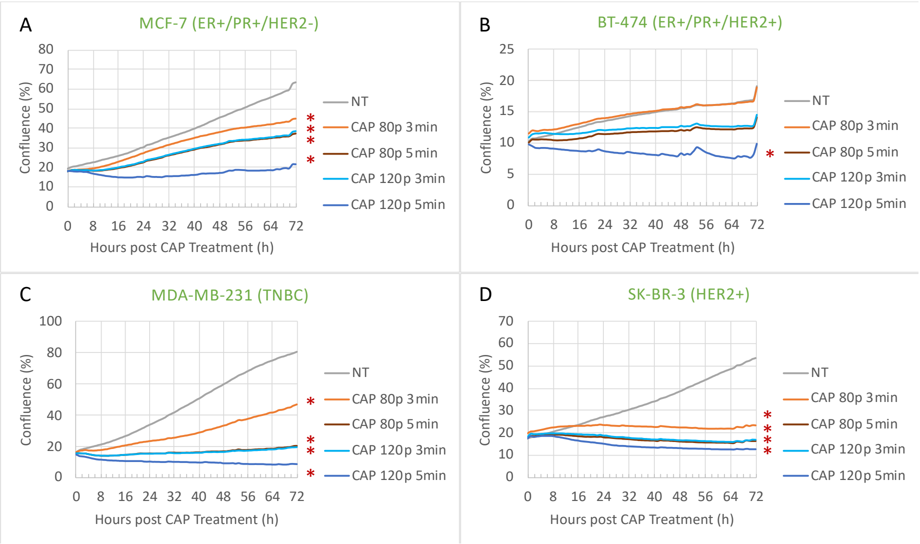

Supplementary Fig. S1 Confluence of (A) MCF-7, (B) BT-474, (C) MDA-MB-231, and (D) SK-BR-3 cells with or without CHCP treatment over 72 hours. Student t test was performed on each treatment dosage and every hour post CAP treatment compared to NT (* $p < 0.05$).

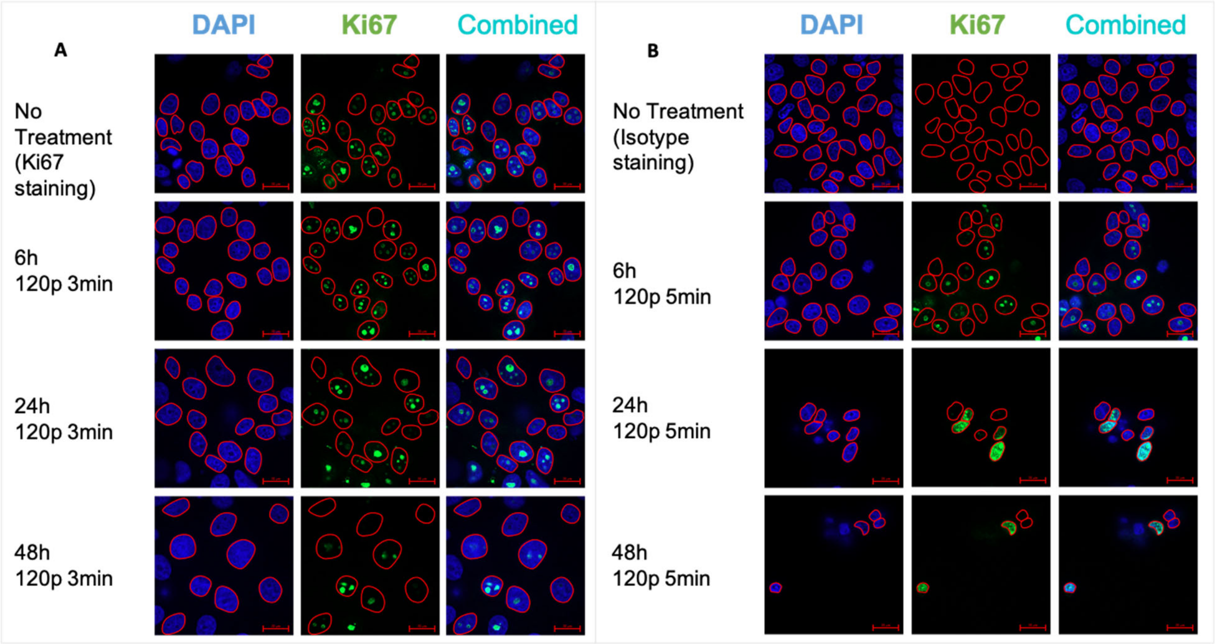

Supplementary Fig. S2 Ki67 staining of MCF-7 ($ER^+PR^+HER2^-$) cell line. A) Representative confocal microscopic images of MCF-7 cells 6/24/48 h post-CAP treatment at 120p for 3min (scale bar 50 μm). B) Representative confocal microscopic images of MCF-7 cells 6/24/48 h post-CAP treatment at 120p for 5min (scale bar 50 μm).

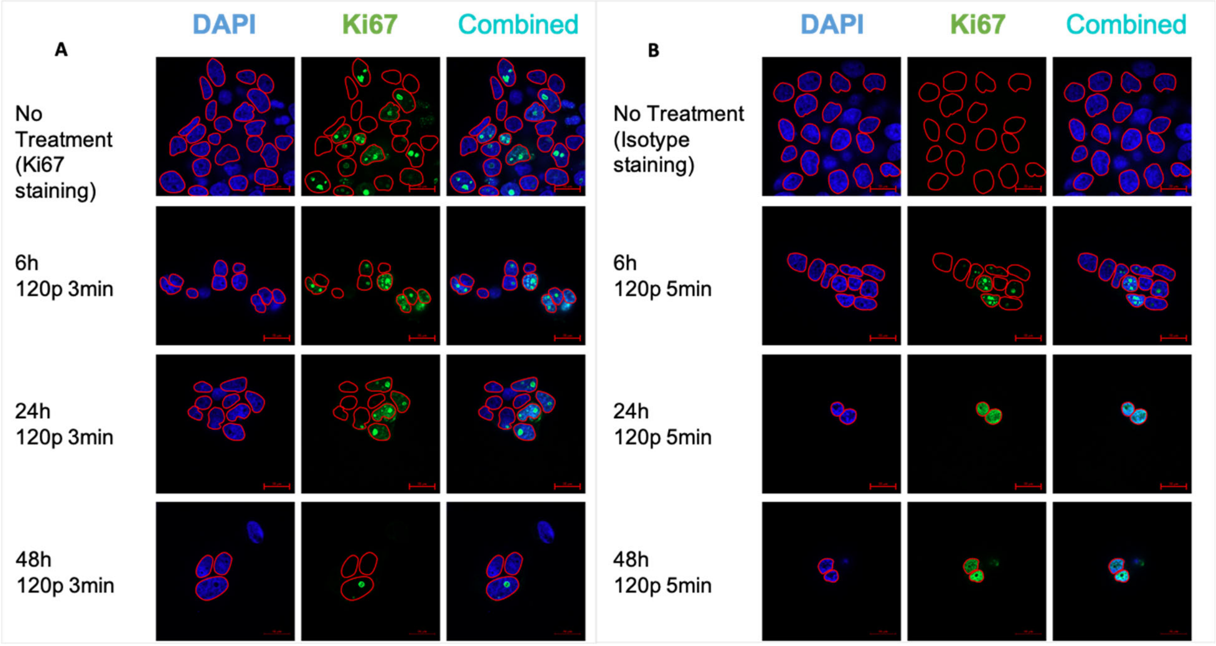

Supplementary Fig. S3. Ki67 staining of BT-474 ($ER^+PR^+HER2^+$) cell line. A) Representative confocal microscopic images of BT-474 cells 6/24/48 h post-CAP treatment at 120p for 3min (scale bar 50 μm). B) Representative confocal microscopic images of BT-474 cells 6/24/48 h post-CAP treatment at 120p for 5min (scale bar 50 μm).

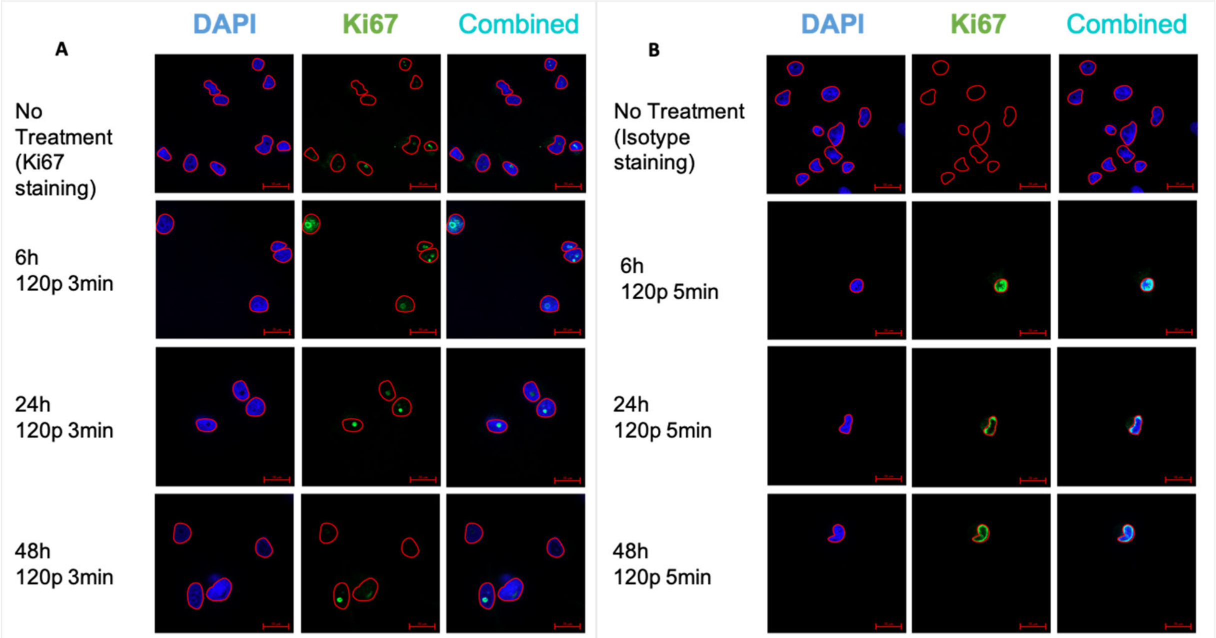

Supplementary Fig. S4 Ki67 staining of MDA-MB-231 (TN) cell line. A) Representative confocal microscopic images of MDA-MB-231 cells 6/24/48 h post-CAP treatment at 120p for 3min (scale bar 50 μm). B) Representative confocal microscopic images of MDA-MB-231 cells 6/24/48 h post-CAP treatment at 120p for 5min (scale bar 50 μm).

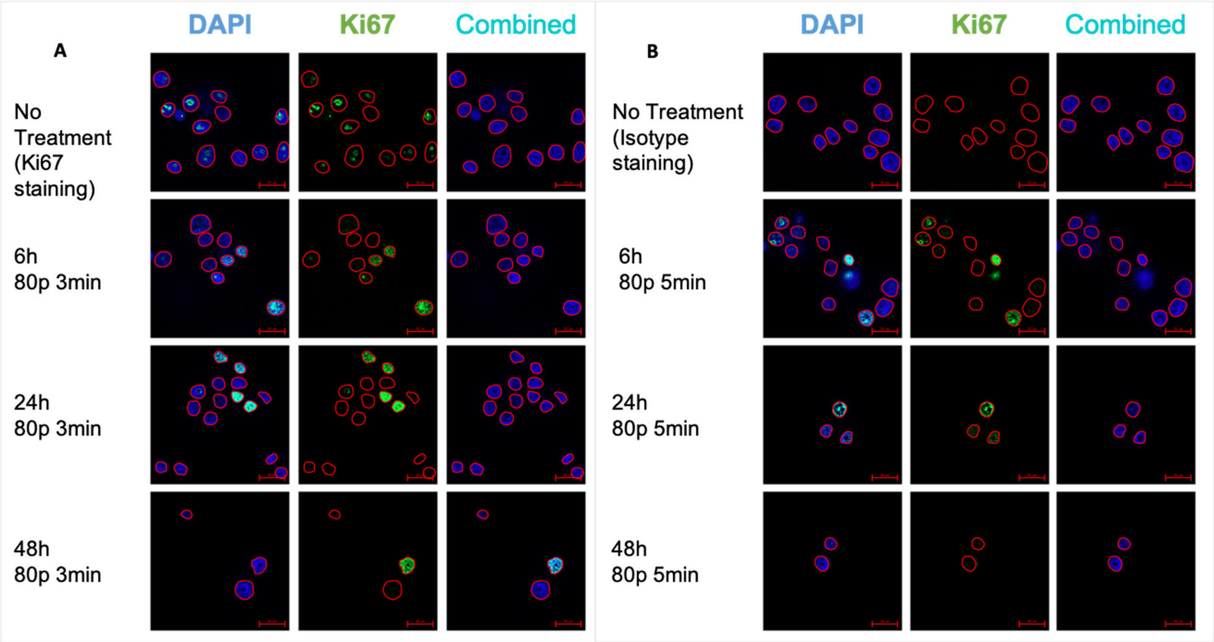

Supplementary Fig. S5 Ki67 staining of SK-BR-3 ($HER2^+$) cell line. A) Representative confocal microscopic images of SK-BR-3 cells 6/24/48 h post-CAP treatment at 80p for 3min (scale bar 50 μm). B) Representative confocal microscopic images of SK-BR-3 cells 6/24/48 h post-CAP treatment at 80p for 5min (scale bar 50 μm).

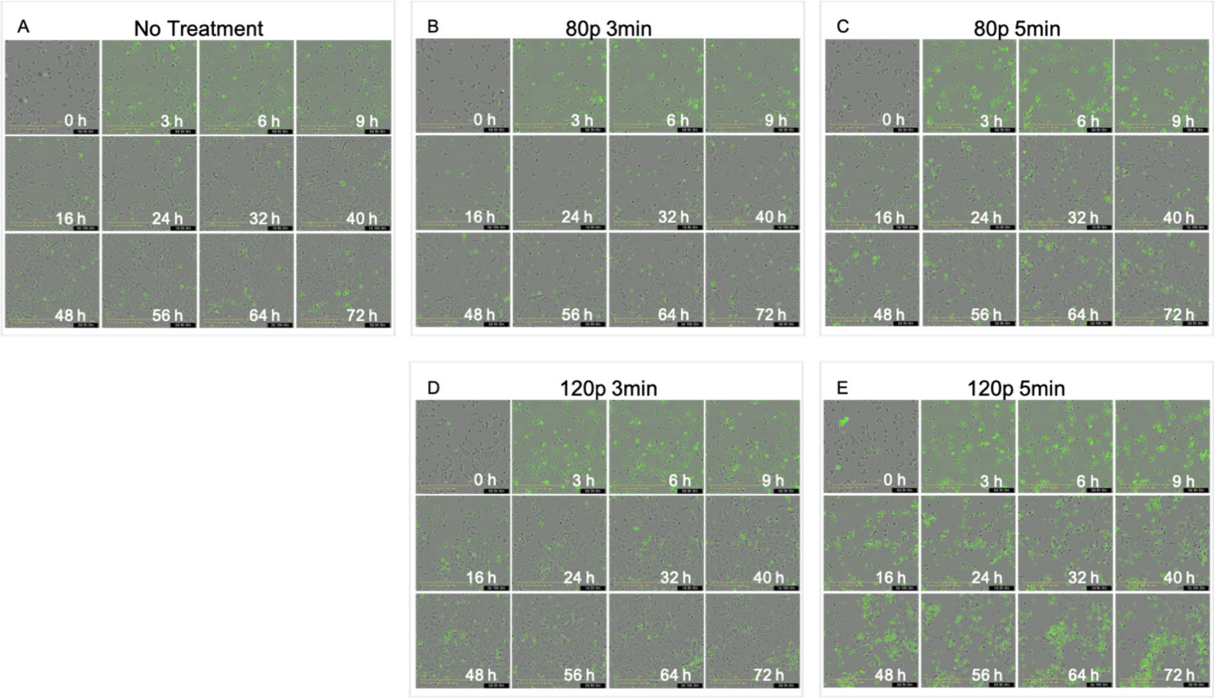

Supplementary Fig. S6 Phase contrast images of MCF-7 ($ER^+PR^+HER2^-$) cell line stained with Incucyte® Caspase-3/7 Dyes for Apoptosis (green). A-E) Representative images of MCF-7 cells 0-72 h post-CAP treatment at 80p or 120p for 3min or 5min (scale bar 400 μm).

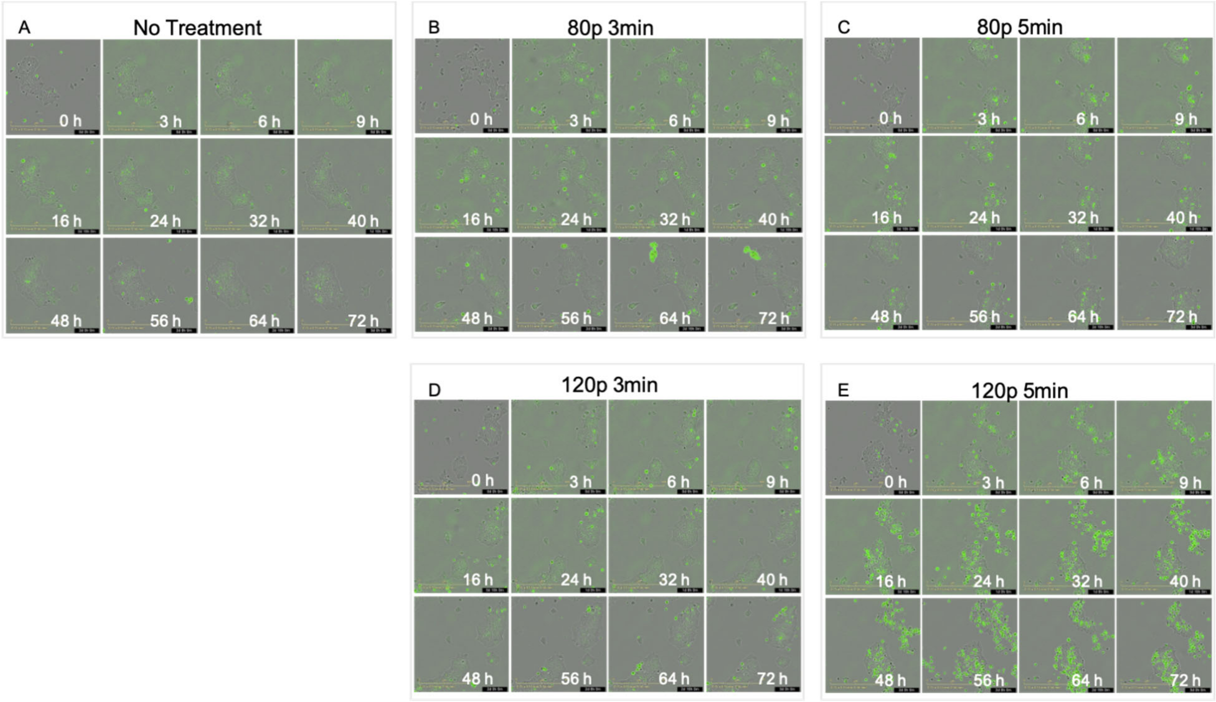

Supplementary Fig. S7 Phase contrast images of BT-474 ($ER^{+}PR^{+}HER2^{+}$) cell line stained with Incucyte® Caspase-3/7 Dyes for Apoptosis (green). A-E) Representative images of MCF-7 cells 0-72 h post-CAP treatment at 80p or 120p for 3min or 5min (scale bar 400 μm).

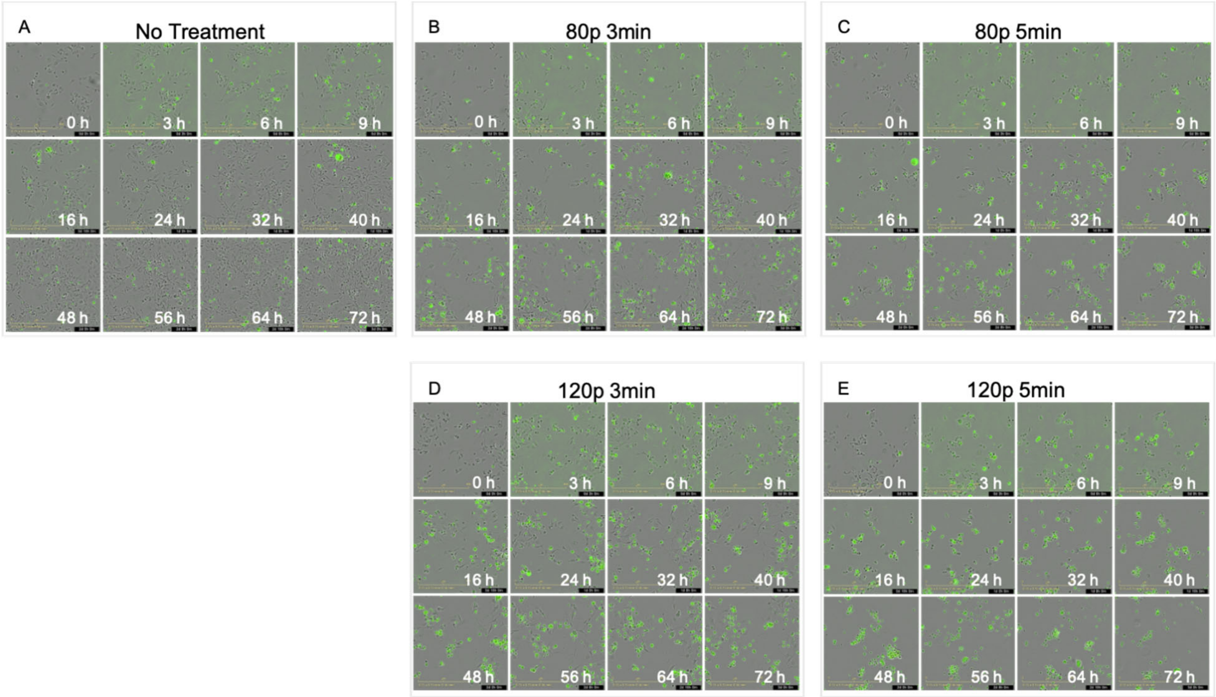

Supplementary Fig. S8 Phase contrast images of MDA-MB-231 (TNBC) cell line stained with Incucyte® Caspase-3/7 Dyes for Apoptosis (green). A-E) Representative images of MCF-7 cells 0-72 h post-CAP treatment at 80p or 120p for 3min or 5min (scale bar 400 μm).

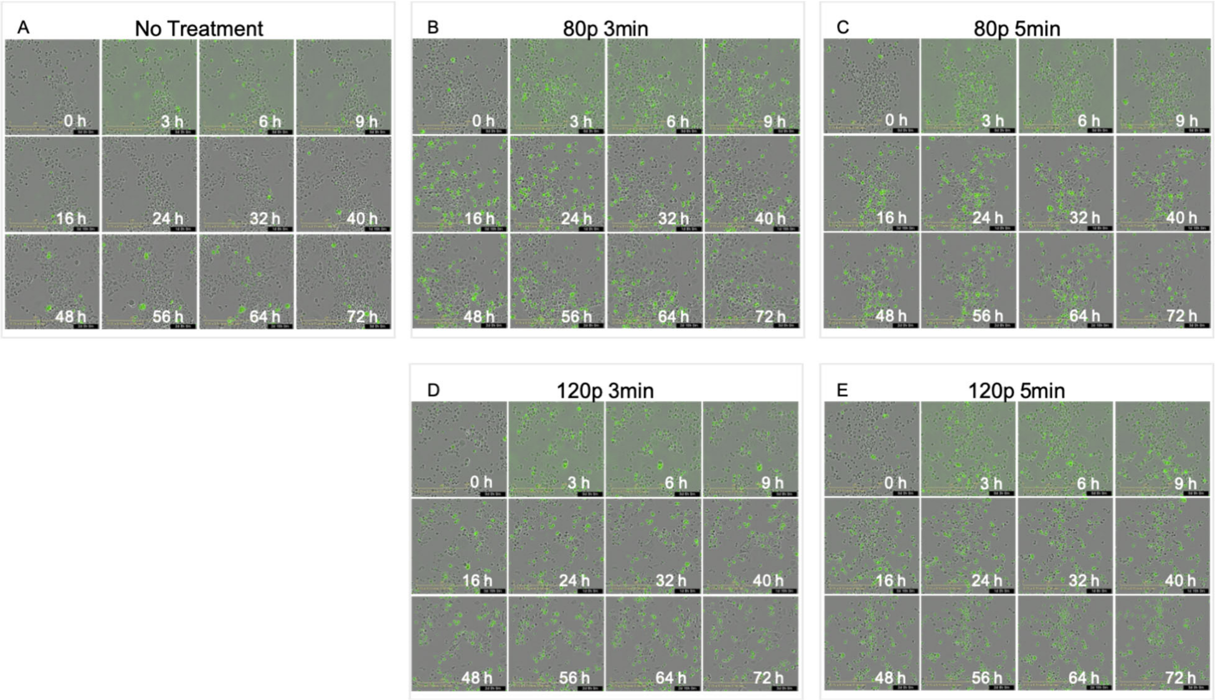

Supplementary Fig. S9 Phase contrast images of SK-BR-3 (ER$^-$PR$^-$HER2$^+$) cell line stained with Incucyte® Caspase-3/7 Dyes for Apoptosis (green). A-E) Representative images of MCF-7 cells 0-72 h post-CAP treatment at 80p or 120p for 3min or 5min (scale bar 400 μm).

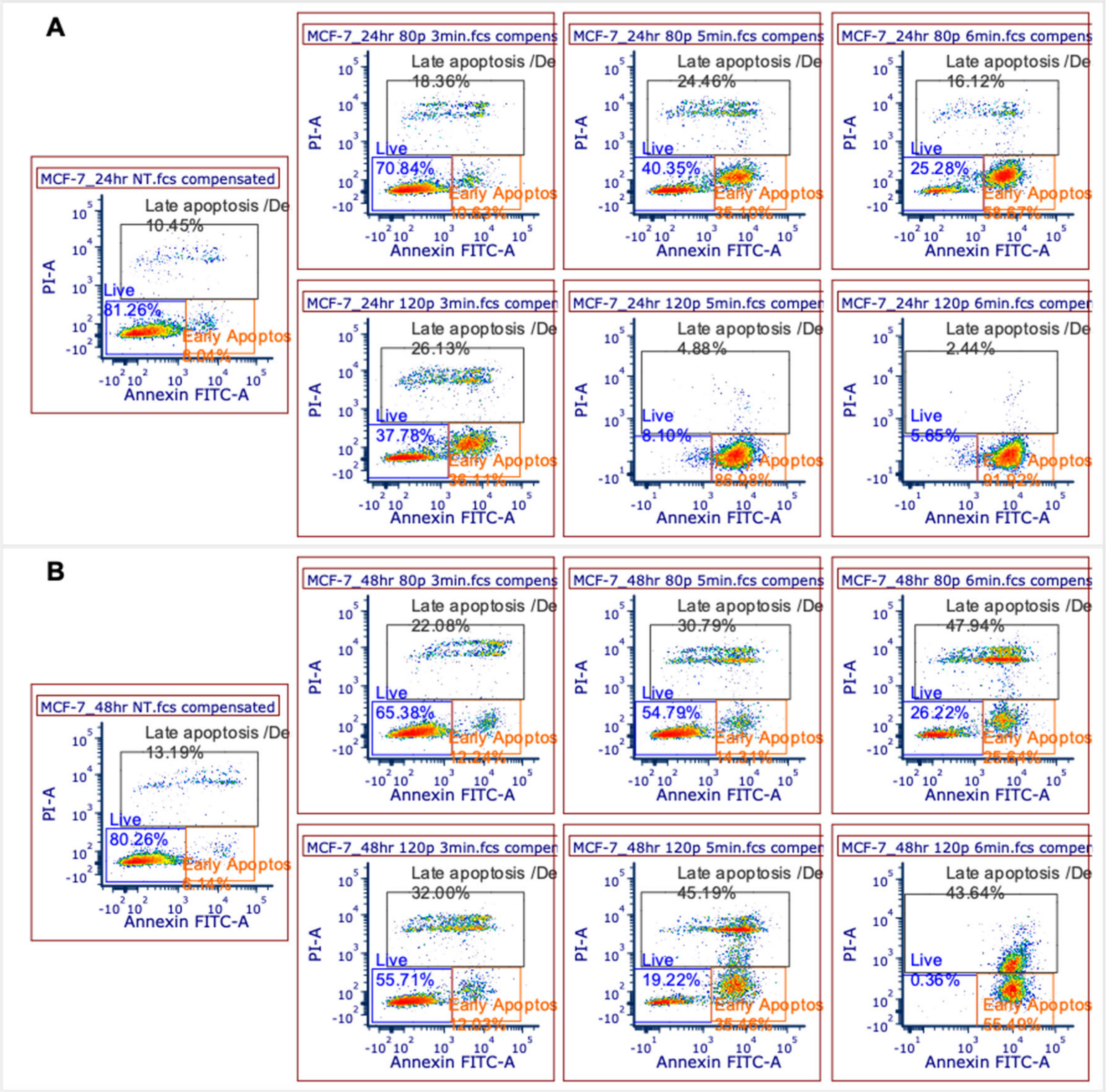

Supplementary Fig. S10 Apoptosis analysis of MCF-7 ($ER^{+}PR^{+}HER2^{-}$) cell line. A) Representative scatter plot of MCF-7 cells stained by Annexin V vs. PI from one experiment 24 h post CAP treatment. B) Representative scatter plot of MCF-7 cells stained by Annexin V vs. PI from the same experiment 48 h post CAP treatment.

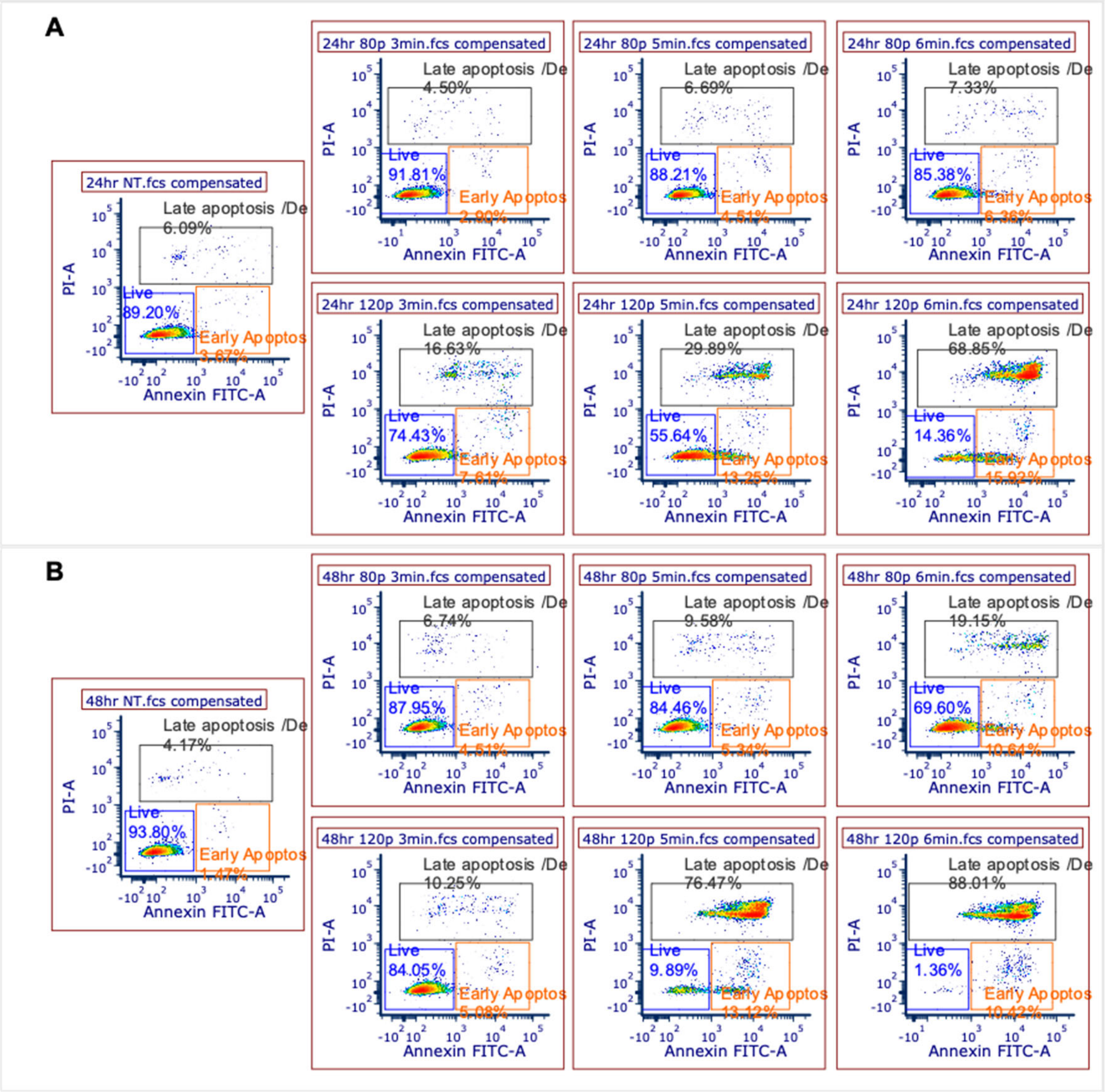

Supplementary Fig. S11 Apoptosis analysis of BT-474 ($ER^+PR^+HER2^+$) cell line. A) Representative scatter plot of BT-474 cells stained by Annexin V vs. PI from one experiment 24 h post CAP treatment. B) Representative scatter plot of BT-474 cells stained by Annexin V vs. PI from the same experiment 48 h post CAP treatment.

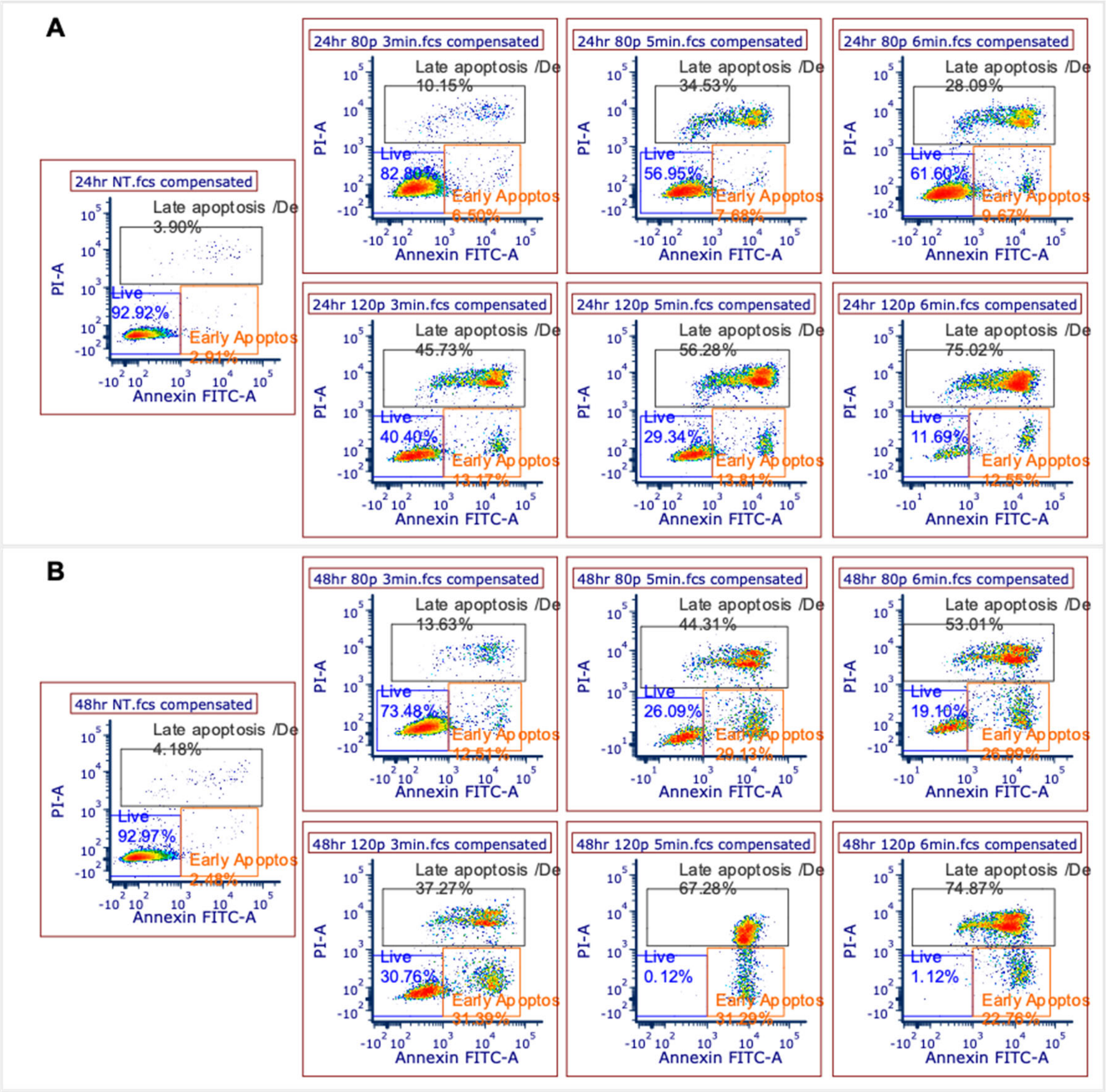

Supplementary Fig. S12 Apoptosis analysis of MDA-MB-231 (TN) cell line. A) Representative scatter plot of MDA-MB-231 cells stained by Annexin V vs. PI from one experiment 24 h post CAP treatment. B) Representative scatter plot of MDA-MB-231 cells stained by Annexin V vs. PI from the same experiment 48 h post CAP treatment.

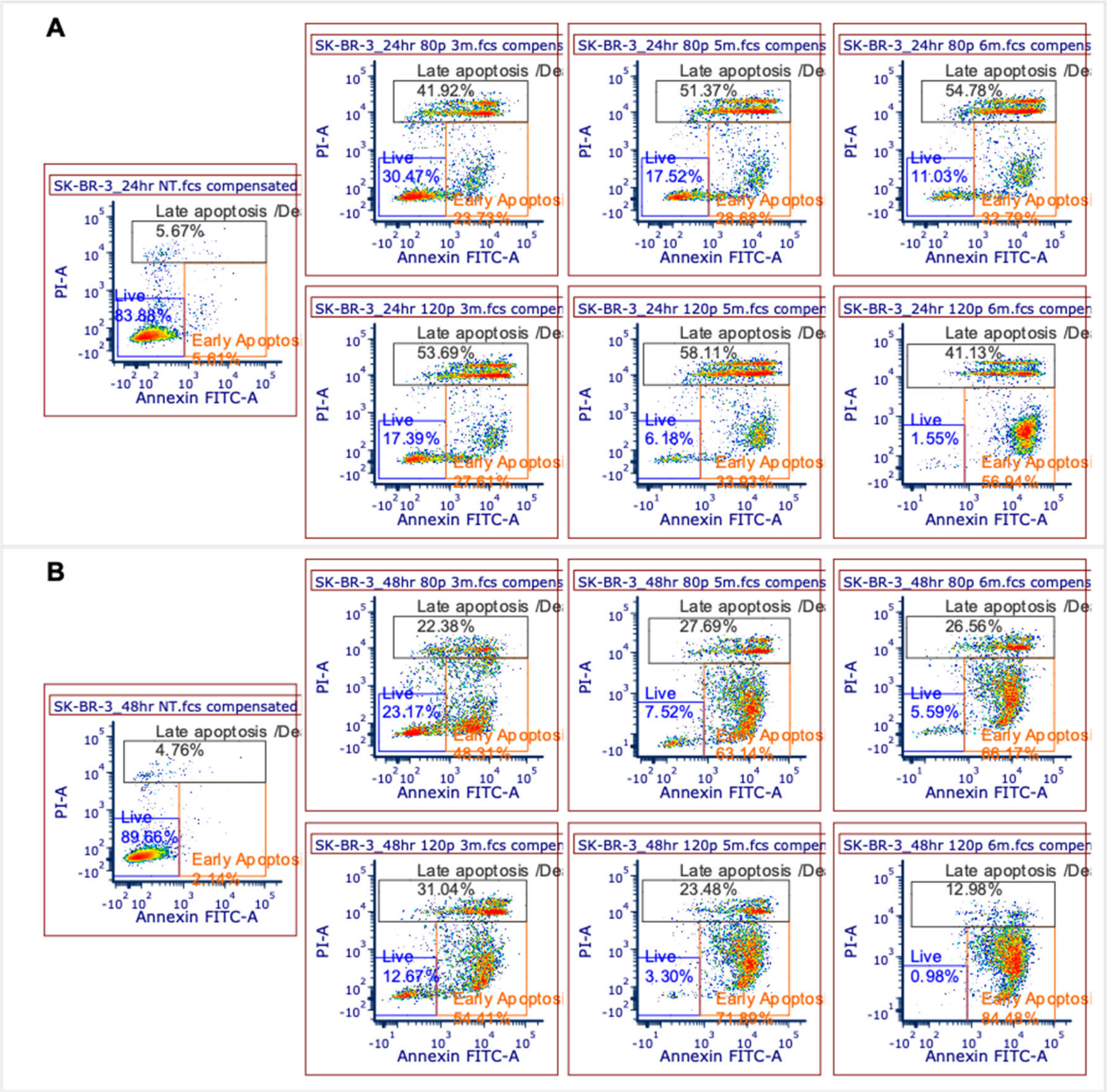

Supplementary Fig. S13 Apoptosis analysis of SK-BR-3 (HER2[+]) cell line. A) Representative scatter plot of SK-BR-3 cells stained by Annexin V vs. PI from one experiment 24 h post CAP treatment. B) Representative scatter plot of SK-BR-3 cells stained by Annexin V vs. PI from the same experiment 48 h post CAP treatment.

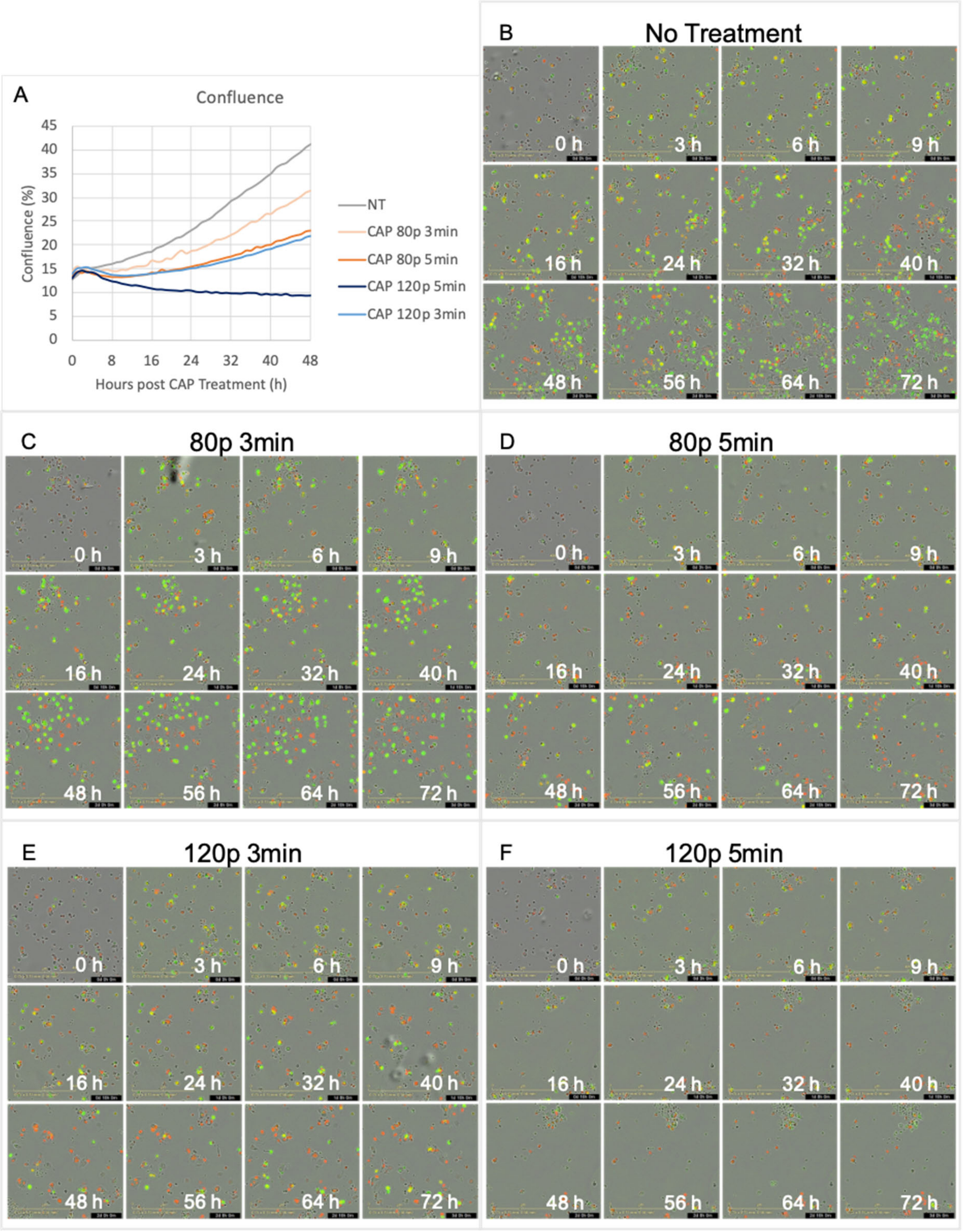

Supplementary Fig. S14 Cell cycle of MCF-7 (ER$^+$PR$^+$HER2$^-$) stable cell line generated with IncuCyte® Cell Cycle Green/Red Lentivirus Reagent untreated or treated by CAP at 80p or 120p for 3min or 5min. A) Confluence of cells 0-48 hours post treatment B-F) Representative phase contrast images 0-72 h post-CAP treatment (scale bar 400 μm).

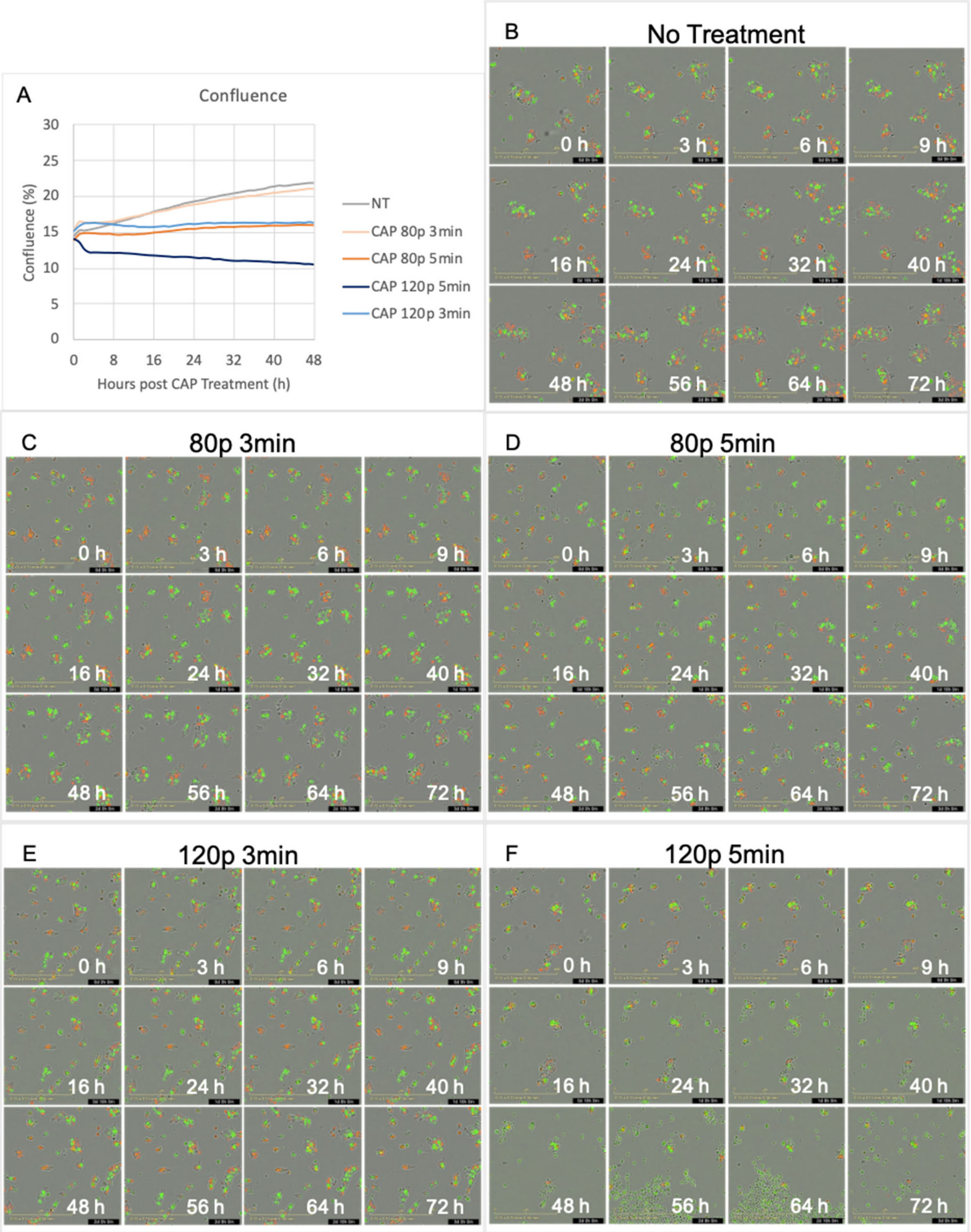

Supplementary Fig. S15 Cell cycle of BT-474 (ER$^+$PR$^+$HER2$^+$) stable cell line generated with IncuCyte® Cell Cycle Green/Red Lentivirus Reagent untreated or treated by CAP at 80p or 120p for 3min or 5min. A) Confluence of cells 0-48 hours post treatment B-F) Representative phase contrast images 0-72 h post-CAP treatment (scale bar 400 μm).

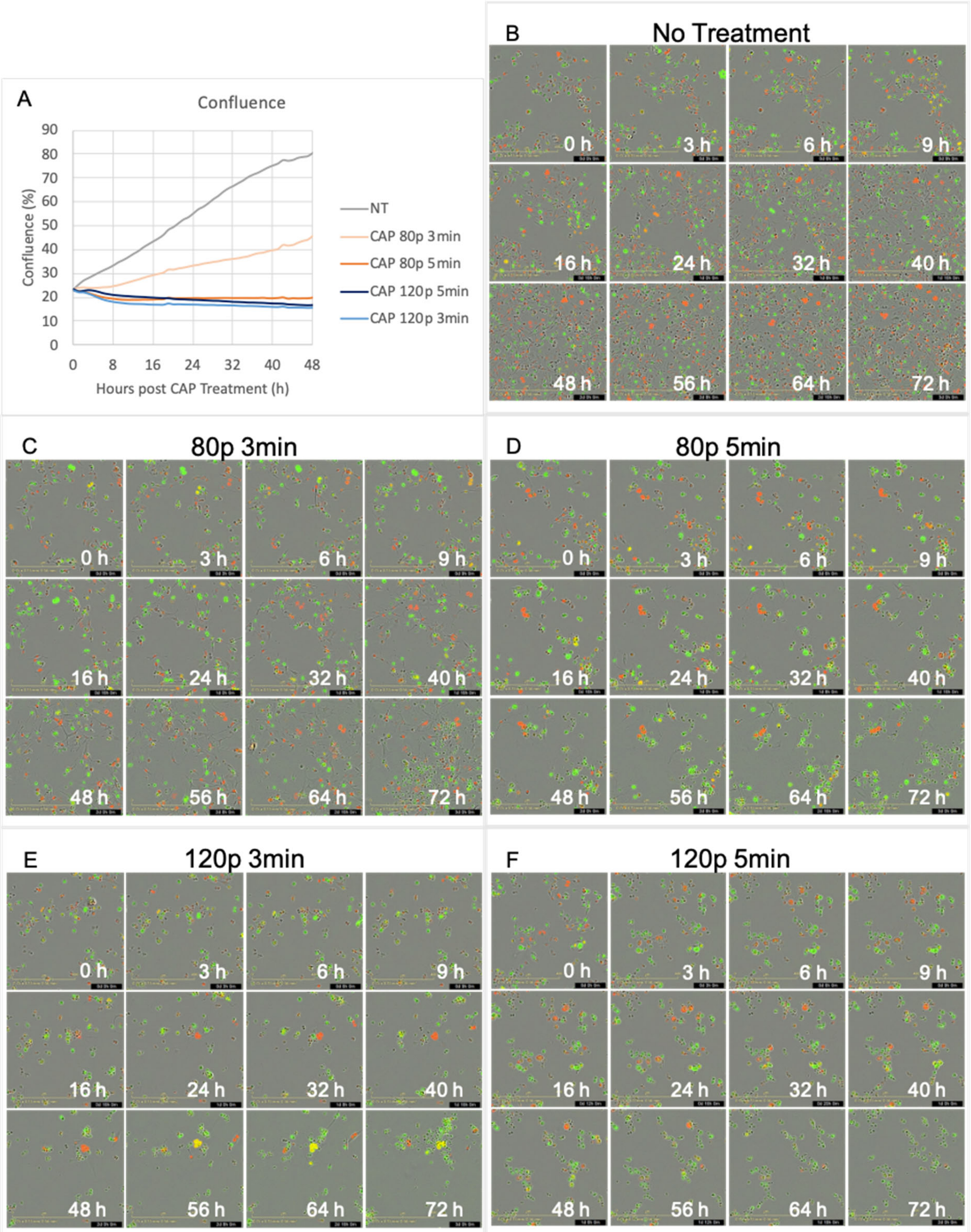

Supplementary Fig. S16 Cell cycle of MDA-MB-231 (TNBC) stable cell line generated with IncuCyte® Cell Cycle Green/Red Lentivirus Reagent untreated or treated by CAP at 80p or 120p for 3min or 5min. A) Confluence of cells 0-48 hours post treatment B-F) Representative phase contrast images 0-72 h post-CAP treatment (scale bar 400 μm).

Samples & Histone genes	CAP 0hr vs Mock 0 hr	CAP 1hr vs Mock 1 hr	CAP 2 hr vs Mock 2 hr	CAP 3 hr vs Mock 3 hr	CAP 0 hr vs CAP 1 hr	CAP 0 hr vs CAP 2 hr	CAP 0 hr vs CAP 3 hr	CAP 1 hr vs CAP 2 hr	CAP 1 hr vs CAP 3 hr	CAP 2 hr vs CAP 3 hr
HIST1H1C			*	*		*	*			
HIST1H2AB		*	*	*	*	*	*			
HIST1H2AC		*	*	*	*	*	*			
HIST1H2AG			*	*		*	*			
HIST1H2AI		*	*	*	*	*	*			
HIST1H2BJ	*	*	*	*	*	*	*			
HIST1H2BK		*	*	*	*	*	*			
HIST1H2BN		*	*	*	*	*	*			
HIST1H2BO		*	*	*	*	*	*			
HIST1H3A		*	*	*	*	*				
HIST1H3B		*	*	*	*	*	*			
HIST1H3C										
HIST1H3H			*	*		*	*			
HIST1H4B		*		*	*		*			
HIST2H3D		*	*	*	*	*	*			
HIST4H4	*		*	*						

Supplementary Fig. S17 Histone RNA degradation after CAP treatment statistical analysis. Repeated measures ANOVAs was performed followed by post-hoc comparisons using Student *t* test with Bonferroni corrections as appropriate. *P<0.005.

Histone Genes	1H2AB	1H2AC	1H2AI	1H2BJ	1H2BK	1H2BN	1H2BO	1H3A	1H3B	1H3C	1H3H	1H4B	2H3D	4H4
t Test with Bonferroni Correction														
CAP-ZERO-HRS-IP Vs MOCK-ZERO-HRS-IP	*	*	*	*	*	*	*	*	*	*	*	*	*	*
CAP-ONE-HRS-IP Vs MOCK-ONE-HRS-IP	*	*	*	*	*	*	*	*	*		*	*	*	*
CAP-ZERO-HRS-IP Vs MOCK-ZERO-HRS-IN	*	*	*	*		*	*	*	*	*	*	*	*	
CAP-ONE-HRS-IP Vs MOCK-ONE-HRS-IN	*	*	*	*				*	*	*	*	*	*	*
CAP-ZERO-HRS-IP Vs CAP-ONE-HRS-IP					*	*	*							

Supplementary Fig. S18 Pull-down of 8-oxoG Histone RNA statistical analysis. Repeated measures ANOVAs was performed followed by post-hoc comparisons using Student *t* test with Bonferroni corrections as appropriate. *P<0.005.
